# Optimization of tick identification via MALDI-TOF MS for implementation in routine diagnostics

**DOI:** 10.1007/s00436-026-08769-0

**Published:** 2026-09-25

**Authors:** Maximilian Münch, Andreas Wieser, Christof Geldmacher, Stefan Kammermeier, Reinhard K. Straubinger, Ard M. Nijhof, Thomas Maier, Lidia Chitimia-Dobler

**Affiliations:** 1 Fraunhofer Institute for Translational Medicine and Pharmacology ITMP, Immunology, Infection and Pandemic Research IIP, Türkenstraße 87, 80799 Munich, Germany; 2 Institute of Infectious Diseases and Tropical Medicine, LMU University Hospital, Munich, Germany; 3 German Center for Infection Research (DZIF), Partner Site Munich, Munich, Germany; 4 Department of Neurology, University Hospital LMU, Munich, Germany; 5 Chair of Microbiology and Mycology, Department of Veterinary Sciences, Faculty of Veterinary Medicine, LMU, Munich, Germany; 6 Institute of Parasitology and Tropical Veterinary Medicine, Freie Universität Berlin, Berlin, Germany; 7 Veterinary Center for Resistance Research, Freie Universität Berlin, Berlin, Germany; 8 Bruker Daltonics GmbH & Co. KG, Bremen, Germany; 9 Bundeswehr Institute of Microbiology, Munich, Germany; 10 Chair of Experimental Parasitology, Department of Veterinary Sciences, Faculty of Veterinary Medicine, LMU, Munich, Germany

**Keywords:** Ixodid tick identification, MALDI-TOF MS, Biotyper®, Protein extraction, Protein fingerprinting

## Abstract

Ticks are important vectors of pathogens with substantial medical and veterinary relevance, a threat that may be further increased with the spread of invasive tick species. Reliable tick identification is relevant for accurate diagnostic elucidation of transmission pathways, and the development of targeted surveillance strategies and risk assessments. A standardized, rapid, and cost-effective identification workflow that can be implemented across laboratories is needed. Previous studies have shown that MALDI-TOF MS is a promising, objective, and economical approach for tick identification. However, the methodology remains insufficiently standardized and is not yet routinely applied for vector identification. The aim of this study was therefore to optimize MALDI-TOF MS-based tick identification for routine use in diagnostic laboratories. A simplified and standardized protocol for protein extraction from whole tick legs via brief incubation in 70% formic acid is presented. This protocol provides a robust basis for the development of a curated MALDI Biotyper® reference library, enabling reliable, expert-independent tick identification and supporting epidemiological surveillance of vectors and vector-borne pathogens.

## Introduction

Ticks are major vectors of pathogens affecting both humans and animals and are therefore of substantial medical and veterinary importance (Jongejan and Uilenberg 2004; Smith 2005; Sonenshine and Roe 2014). Some pathogens are associated specifically with one genus, e.g., *Borrelia burgdorferi* sensu lato with tick species from the genus *Ixodes* (*I. ricinus* and *I. persulcatus* in Eurasia and *I. scapularis* and *I. pacificus* in North America (Stanek et al. 2012)). Accurate tick identification is therefore relevant for an accurate diagnostic evaluation, elucidation of transmission pathways, and the development of targeted surveillance strategies and evidence-based risk assessments. Although morphological identification may remain the conventional reference standard, its practical utility has limitations. The inherent subjectivity of morphological criteria, combined with the declining availability of trained taxonomists, can create critical bottlenecks for expensive monitoring programs. Even when expertise is available, reliable identification may be hindered by engorged, damaged or incomplete specimens and is particularly difficult for immature developmental stages (Parola and Raoult 2001; Jongejan and Uilenberg 2004; de la Fuente et al. 2008; Yssouf et al. 2013; Koffi et al. 2017; Rich et al. 2023; Galletti et al. 2024). In addition, closely related species may be difficult to distinguish morphologically, as illustrated by studies demonstrating that German specimens morphologically identified as *Ixodes inopinatus* were in fact *I. ricinus* based on molecular analysis (Rollins et al. 2023).

Molecular identification, particularly deoxyribonucleic acid (DNA) analysis targeting 16S mitochondrial ribosomal ribonucleic acid (rRNA) gene sequence, offers objective and high-resolution species identification (Black and Piesman 1994; Lv et al. 2014). Given the ubiquity of DNA across tissues, molecular identification can also be performed on damaged specimens that are unsuitable for morphological identification. Despite its precision, this method requires polymerase chain reactions (PCR) and sequencing workflows, the availability of reliable reference sequence databases, dedicated infrastructure, and specialized personnel. These requirements increase cost and turnaround time and limit suitability for high-throughput surveillance activities (Black and Piesman 1994; Yssouf et al. 2016; Dantas-Torres 2018; Jia et al. 2020; Galletti et al. 2024).

Matrix-Assisted Laser Desorption/Ionization Time-of-Flight Mass Spectrometry (MALDI-TOF MS) offers a rapid, cost-effective and objective alternative for species identifications from small amounts of material, making it suitable for high-throughput screening and for specimens that are difficult to classify morphologically. The approach has revolutionized routine microbial diagnostics and is increasingly being applied to arthropod taxonomy by the analysis of species-specific protein fingerprints.

Protein spectra have been used to distinguish mosquitoes, sand flies, and ticks at the species level, with reproducible profiles across multiple laboratories and geographic settings. These results from various vector groups demonstrate the high effectiveness of this approach (Wieser et al. 2012; Sambou et al. 2015; Lafri et al. 2016; Yssouf et al. 2016; Boyer et al. 2017; Vega-Rúa et al. 2018; Benyahia et al. 2021; Galletti et al. 2024; Bouledroua et al. 2025). For ticks, MALDI-TOF MS has shown high concordance with independent taxonomic methods. In Vietnam, blind testing achieved 100% correct species assignments with log(score) values typically between 1.70 and 2.39 (Scale ranging from 0 = “not identified” to 3 = 100% match), indicating reliable discrimination under routine conditions (Huynh et al. 2021). This technology may also resolve closely related taxa, e.g., the differentiation of *Dermacentor variabilis* and the recently described *Dermacentor similis*, underscoring its potential where morphological identification is challenging (Galletti et al. 2024). Importantly, MALDI-TOF mass spectra remain informative even for engorged female ticks, which are often difficult to identify morphologically due to body distention, thereby supporting field applicability and retrospective analyses (Huynh et al. 2021).

Despite these promising studies, MALDI-TOF MS has not yet been widely standardized or implemented for routine vector identification. One reason is the lack of available standardized, curated, validated, well-maintained and up-to-date reference databases for comparing unknown samples, although open community platforms exist. In addition, many existing sample preparation protocols are unsuitable for routine diagnostic workflows. Beltran et al. (2023) have initiated efforts to transfer tick identification from the research settings to routine diagnostic labs, underscoring the relevance of tick identification in medicine.

To be economical for commercial routine diagnostic laboratories, reliable tick identification methods must be simple, robust and compatible with existing laboratory resources and reagents, economical and quick to validate, while offering reproducible and validated performance across laboratories (Wieser et al. 2012). However, effective and established protein extraction methods and sample preparation protocols often involve laborious protocols and/or hazardous substances such as liquid nitrogen, acetonitrile, acetone or special ball mills (Damerval et al. 1986; Boucheikhchoukh et al. 2018; Boyer et al. 2019; Neumann-Cip et al. 2020; Galletti et al. 2024). The present study therefore aimed to establish a simple, reliable and cost-effective workflow for protein extraction and sample preparation workflow by adapting and optimizing different techniques and existing protocols for tick identification using MALDI-TOF MS.

## Materials and methods

### Tick origin and identification

Tick specimens (males & females) were collected by flagging [*I. ricinus* (nymphs and adults) and *Dermacentor reticulatus*] or originated from tick colonies at the Institute for Parasitology and Tropical Veterinary Medicine of the Freie Universität Berlin [*Hyalomma dromedarii* and *I. ricinus* (adults)]. *Ixodes persulcatus* ticks were acquired from a commercial tick colony (Insect Service, Berlin, Germany). All ticks were stored up to six months in 70% ethanol [≥ 99.8% - HPLC grade - VWR International GmbH, Darmstadt, Germany]/30% H_2_O [HPLC grade - VWR International GmbH] (v/v). Some preliminary approaches were tested using ticks stored in ≥ 99.8% ethanol [HPLC grade].

All specimens were identified using standard taxonomic keys and morphological characteristics including palpal structures, scutal patterns, spiracular plates, and genital apertures (Apanaskevich et al. 2008; Pérez-Eid 2009). High-resolution digital microscopy images were taken with either a Keyence VHX-900F microscope (Itasca, IL, USA) or a LEICA M205 C (Leica Microsystems GmbH, Wetzlar, Germany).

DNA extractions were performed from five randomly chosen tick bodies according to Wiesinger et al. (2023), after leg removal for MALDI-TOF MS analysis using a Promega Maxwell® Instrument with Maxwell® RSC Blood DNA Kit [Promega GmbH, Walldorf, Germany]. Molecular identification was performed by amplifying an approximately 460-bp fragment of the mitochondrial 16S rRNA gene using the primers 16S + 1 (5′‑ CTG CTC AAT GAT TTT TTA AAT T ‑3′) and 16S − 1 (5′‑ CCG GTC TGA ACT CAG AT ‑3′) described by described by Black and Piesman (1994) which were slightly shortened. Reactions were carried out with the Q5® High‑Fidelity PCR Kit [New England Biolabs GmbH, Frankfurt am Main, Germany] in a total volume of 20 µl containing 1 µl template DNA, 1 µl (0.5 µM) of each primer, 7 µl of nuclease-free water and 10 µl of Q5 High-Fidelity 2 × Master Mix, using the following cycling conditions: initial denaturation at 98 °C for 30 s; 36 cycles of 98 °C for 10 s, 52 °C for 30 s and 72 °C for 20 s; and a final extension at 72 °C for 2 min. Amplicons were verified by electrophoresis on a 1% agarose gel, purified with the Promega Wizard® SV Gel and PCR Clean‑Up System [Promega GmbH, Walldorf, Germany] and bidirectionally sequenced with the BigDye™ Terminator v3.1 Cycle Sequencing Kit [Applied Biosystems, San Francisco, CA, USA]. Sequences were trimmed and assembled in SnapGene [GSL Biotech LLC, San Diego, USA] and compared with the NCBI Nucleotide collection (nt) using BLASTn (megablast, default parameters) (Zhang et al. 2000). Species assignment required ≥ 99% nucleotide identity and ≥ 99% query coverage with a conspecific reference sequence, with a clear margin to the next non‑conspecific hit. At least five randomly selected specimens per species were sequenced. The obtained sequences were deposited in GenBank under the following accession numbers: PZ811701—PZ811720.

### MALDI-TOF MS methodology

#### Preliminary approaches tested for the tick leg protein extraction

In a preliminary experimental approach, published protocols with internal adaptations or own approaches were systematically studied by visual inspection of the resulting spectra (Table 1). The resulting spectra, the workload/cost to result ratio (efficiency) and especially the feasibility for diagnostic labs were weighed against one another for the development of the final method. Feasibility for diagnostic implementation was defined as compatibility with standard equipment and reagents already established in routine diagnostic laboratories, avoiding specialized devices (e.g., ball mills) and any additional new chemicals or instruments to avoid additional expenses. The preliminary approaches listed in Table 1 were performed as exploratory experiments during method establishment and were therefore assessed qualitatively; hands‑on times were recorded only for the three complete workflows that were subsequently compared directly with one another.

**Table 1:** Preliminary approaches tested in the early stage for the establishment of a novel tick leg protein extraction method

Homogenization Methods		Adapted from
	H1: Homogenization of the legs in the solvents with a plastic micro pestle by hand at room temperature or with a drilling machine at low speed on ice (tested with E1-3, SP1 and E1-4, SP2)	(Rothen et al. 2016; Beltran et al. 2023)
	H2: Ultrasonic bath for 10 min on ice (tested with H1, E1, E1a, E1b, SP1; E1, E1a, E1b, H6, SP3)	(Choi et al. 2017)
	H3: Physical pulverization with steel-balls (3 mm) under use of liquid nitrogen and a Vortex. (ball mill gave similar small fragments) (tested with E1, SP1)	(Ericsson and Nistér 2011; Nebbak et al. 2017; Boyer et al. 2019; Galletti et al. 2024; Retsch GmbH 2025)
	H4: Physical pulverization with steel-balls (3 mm) under use of dry ice (CO_2_) and a Vortex. (tested with E1, SP1; 3 min incubation time)	(Ericsson and Nistér 2011; Nebbak et al. 2017; Boyer et al. 2019; Galletti et al. 2024; Retsch GmbH 2025)
	H5: Tick legs with chitinase enzyme [Merck, Germany, No. C6137] in H_2_O or KP_i_ buffer (10 min or 24 h incubation at 37 °C), followed by a Formic Acid (FA) extraction (tested with H1, SP1, E1 and H6, SP3, E1)	This study
	H6: No homogenization (just incubation with extraction solvents) (tested with E1, SP3; with 1, 3, 5, 10, and 15 min incubation time and E1a-4, SP3; with 3 min incubation time)	This study
Extraction Solutions	E1: FA (70%), H_2_O (30%), (v/v) E1a: FA (90%), H_2_O (10%), (v/v) E1b: FA (100%), H_2_O (0%), (v/v)	(Beltran et al. 2023) This study
	E2: 50% Acetonitrile, 35% FA, 15% H_2_O (v/v)	(Neumann et al. 2019; Neumann-Cip et al. 2020)
	E3: Acetonitrile (75%), H_2_O (25%), (v/v), followed by adding FA (75%), dest. H_2_O (25%), (v/v) separately. Same in reverse order was also tested	(Idelevich et al. 2023) This study
	E4: 100% Acetone (−20 °C)/Standard protein precipitation protocol—pelletation, evaporation and reuptake in 75% FA as solvent	(Barritault et al. 1976; Neumann et al. 2019; Neumann-Cip et al. 2020)
Sample Purification	SP1: PTFE Filter Membrane (hydrophilic) 0.2 µm or 0.45 µm	(Neumann et al. 2019; Neumann-Cip et al. 2020) This study
	SP2: Centrifugation at 20,000xG for 3 min	(Rothen et al. 2016; Boyer et al. 2019; Beltran et al. 2023; Galletti et al. 2024)
	SP3: none	This study

Approaches were labeled by order and category as H1–6 for homogenization, E1–4 for extraction, and SP1–3 for sample purification

#### MALDI sample preparation and protein extraction

All ticks were stored up to six months in 70% ethanol and were vortexed in 600 µl H_2_O for 5 s. to remove contaminations. The ticks were subsequently dried on a clean Kimwipe (Kimberly-Clark GmbH, Koblenz, Germany). Tick legs were detached from the idiosoma using a clean fine-tipped forceps [e.g., Carl Roth GmbH + Co. KG, Karlsruhe, Germany (No. 1P2Y.1)] or a pair of fine-tipped scissors and placed at the bottom of a 1.5-ml microcentrifuge Safe-Lock tube (PCR clean; Eppendorf SE, Hamburg, Germany). Forceps were decontaminated between specimens by wiping with 80% isopropanol [≥ 99.9% - HPLC grade - VWR International GmbH]/20% H_2_O [HPLC grade - VWR International GmbH] (v/v). Twenty µl of 70% formic acid (FA) [≥ 99% - analytical reagent - VWR International GmbH]/30% H_2_O [HPLC grade - VWR International GmbH] (v/v) were added to the legs, followed by an incubation for 3 min (maximum 5 min) at room temperature. The liquid phase was subsequently transferred to a new tube and spotted onto the cleaned and heated (35 °C) Bruker MSP 96 polished steel BC target [Bruker Daltonics GmbH & Co. KG, Bremen, Germany]. Target cleaning procedure was done according to the original target cleaning protocol of the manufacturer [Bruker Daltonics GmbH & Co. KG, Bremen, Germany]. For the generation of reference spectra (MSPs), each sample was spotted eight times on a MALDI-TOF MS target. For calibration and as a positive control, 1 µl per spot of Bruker Bacterial Test Standard [Bruker Daltonics GmbH & Co. KG] dissolved in 50 µl Bruker organic solvent for use with MALDI Biotyper® [CHROMANORM® - VWR International GmbH, Germany] was applied. As negative control 1 µl of 70% FA was spotted three times for each measurement run as verification of the cleaning process. Once the sample and control spots were dry (not longer than ten minutes after spotting), they were covered with 1 µl of Bruker HCCA Matrix 2.5 mg [Bruker Daltonics GmbH & Co. KG] dissolved in 250 µl Bruker organic solvent for use with MALDI Biotyper® [CHROMANORM® - VWR International GmbH] using a separate filter pipette tip for each spot.

#### MALDI-TOF MS spectra acquisition

Mass spectrometric analysis was performed with a Bruker MALDI Sirius RUO Biotyper® system (microflex) [Bruker Daltonics GmbH & Co. KG] on Bruker MSP 96 polished steel BC targets [Bruker Daltonics GmbH & Co. KG] with a mass range of 2–20 kDa in linear positive ion mode. The Bruker flexControl Software v. 3.4 [Bruker Daltonics GmbH & Co. KG] was used with the AutoXecute “MBT_AutoX_FilFungi_2” spectra acquisition method, which accumulated up to 800 laser shots per spectrum. The laser repetition rate was 200 Hz and the “spiral small” raster was used. The objective of the method described here was the generation of reference entries of the tick isolates. All eight sample spots were measured three times as recommended by the manufacturer, which resulted in 24 spectra per sample. Before every measurement, the device was calibrated with a BTS (Bacterial Test Standard) [Bruker Daltonics GmbH & Co. KG] spot and a comparable laser power was ensured via a measurement with the “MBT_BTS_Validation_AutoX” acquisition method and a BTS Spot. The laser works according to the manufacturer’s settings if the highest BTS peak is repeatedly and constantly around ~ 20,000–30,000 Intens. [a.u.] (intensity in arbitrary units) when the MBT_BTS_Validation_AutoX method is used manually.

For all shown figures, three biological replicates (ticks) and eight technical replicates (spots) were considered if not stated differently.

#### Spectra processing, quality estimation and recalibration

Raw spectra were processed using the Bruker flexAnalysis v. 3.4 software [Bruker Daltonics GmbH & Co. KG] with default parameters for smoothing and baseline correction. Before evaluation, the control spots (negative: 70% FA & positive: BTS) were checked for validation. Spectral quality was checked for each individual set of spectra. Quality criteria such as the number of peaks, peak intensities, the baseline, the signal-to-noise ratio, peak distribution, and noise were assessed.

All 24 spectra of the same sample (technical replicates) were expected to exhibit consistent characteristics as listed above. If even one of the 24 measured spectra deviated significantly from these quality requirements (e.g., flatline spectrum, significantly lower intensity or higher baseline, missing/additional peaks or oxidation peak artefacts, etc.), it was removed from the spectral set, leaving at least 20 spectra, as recommended by the manufacturer for the creation of MSPs.

**Fig. 1:**
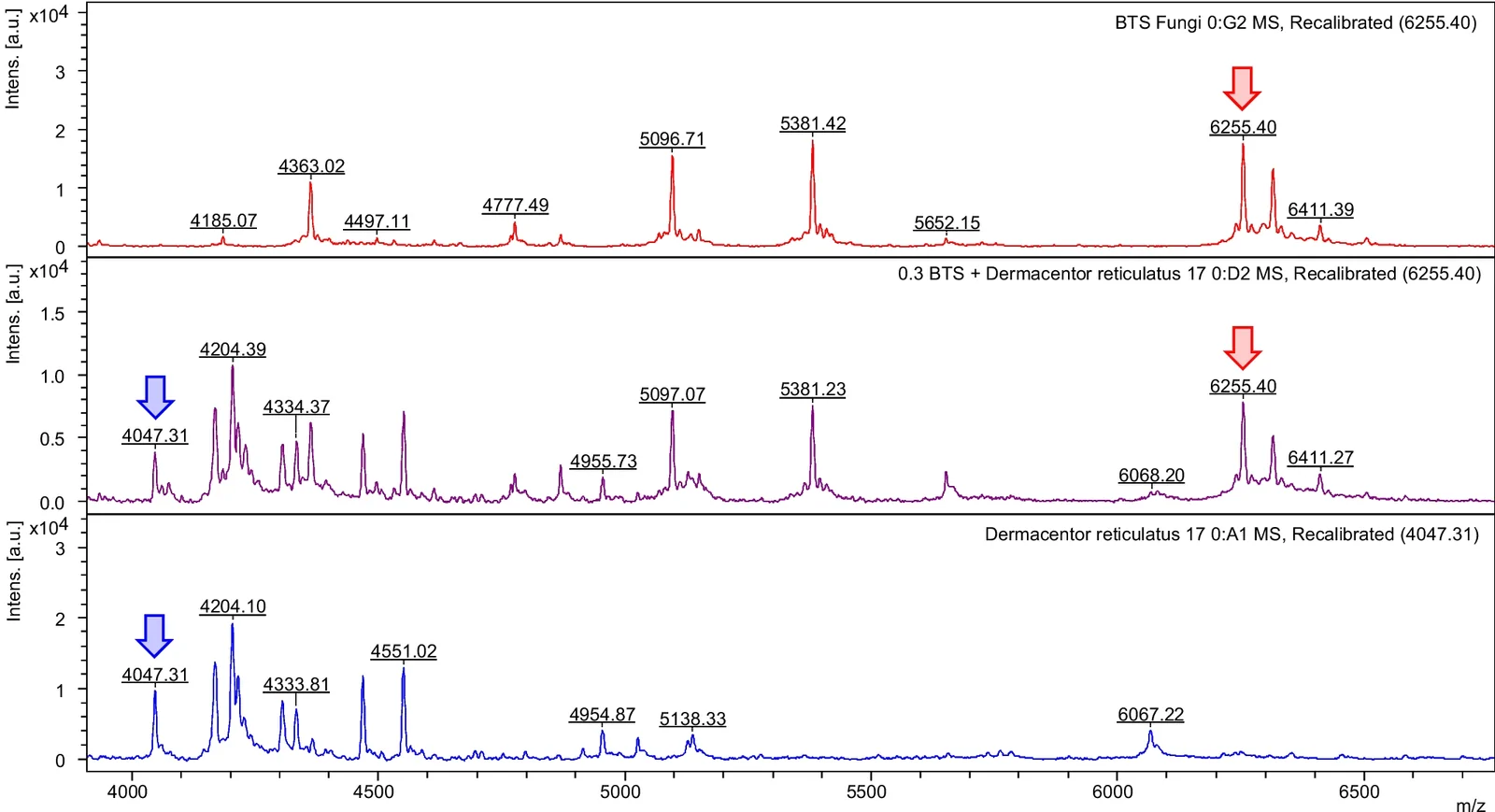
Illustration of the determination of a sample-specific calibration peak. Shown are parts of MALDI-TOF MS spectra of a BTS calibrant (red), the spiked sample (“BTSmix”) with the calibrant (purple) and a tick sample (blue) of a male adult unfed *D. reticulatus* specimen. The red arrow marks the BTS specific peak RS33meth [M + H] + = 6255.4 m/z. This BTS peak was used for single point calibration of the red and the purple spectra. The blue arrow marks the peak with 4047.3 m/z which was used as sample and species-specific calibration peak for the single point calibration of the blue *D. reticulatus* spectra

To prevent and correct for mass shifts and inaccuracies, and bring the reference spectra to the correct masses, recalibrations were performed (see Fig. 1). For each species, at least one additional sample spot (in addition to the 8 spots) was spiked with 0.1 to 1 µl BTS calibration standard and the mixed spectrum was measured accordingly to the other spots (BTSmix spectrum). After the MALDI-TOF MS measurement of all spots, it was checked if the BTSmix spectrum showed the expected mixture of sample peaks and additional BTS peaks. If such individual BTS peaks could be observed and a separation from the sample peaks was approved, a single point calibration with the RS33meth [M + H] + = 6255.4 m/z mass peak of the BTS standard was done. All single point calibration peaks were selected as close as possible to the center of the main mass range (≈ 3000–10000 m/z) of the present sample type (e.g., BTS or a certain species), had to be present with sufficient intensity in all samples of a given sample type and if possible, with no other peaks within ± 1000 ppm to avoid recalibration errors. After the recalibration of the BTSmix spectrum a “typical sample peak” (present in all 24 sample spectra, never present in the pure BTS spectrum) was selected for recalibration of the entire sample spectra set. This sample specific calibration peak needed to be present in all available other biological samples of the same species. The mass of this peak was then used as species specific calibration peak and all spectra of the same species were single point recalibrated with the species-specific calibration peak. Once the species-specific calibration peak was determined, this recalibration process for samples was automated via the Bruker flexAnalysis v. 3.4 Batch Process tool [Bruker Daltonics GmbH & Co. KG] with the “Recalibration_full_process_FA3.4.FAMSMethod” and a respective mass list which contains only the species-specific calibration peak.

Recalibrations were afterwards verified to ensure their correctness e.g., BTS peaks of the BTSmix spectrum and also sample peaks after automatic recalibration were checked and the presence of the calibration peak in all samples of the same species was ensured in order to avoid miscalibration. After that, recalibrated spectra were used for generating reference spectra (MSPs).

#### Creation of reference spectra (MSPs)

After spectra processing, reference spectra - also known as “Main Spectrum Profile (MSP)”- were created with MBT Compass Explorer v. 4.1 [Bruker Daltonics GmbH & Co. KG] out of 20 to 24 spectra per tick isolate as recommended by Bruker with one adaptation: the “BioTyper Preprocessing Standard Method” was slightly changed in the “Mass Adjustment” parameter “Compressing Factor” from 10 to 5. This setting was changed to optimize the proper peak picking especially of peaks in the lower mass range with a close distance to each other.

#### Methods used for performance evaluation of the new sample preparation and protein extraction protocol

All paired protocol comparisons described in this section were performed within single specimens (left four legs vs. right four legs) using unfed adult female *D. reticulatus* collected by flagging, with three biological replicates (*n* = 3) per comparison.

Reproducibility and comparability of spectra between the left and right four legs of the same tick were assessed in three individual biological replicates (three ticks). For each tick, the left four legs and the right four legs were processed separately. MSPs were generated as described above, and pairwise comparisons were performed between the MSPs of the left and right legs from the same tick. Log(scores) were calculated in MALDI Biotyper Compass Explorer v. 4.1 under standard settings, using MSPs from the left legs queried against MSPs from the right legs of the same tick. Resulting log(scores) were averaged over the three replicates, and standard deviations were calculated.

The influence of mechanical homogenization of tick legs on the MALDI-TOF MS spectra was evaluated by comparing extraction with and without homogenization. For each of three ticks, the left legs were processed according to the workflow without homogenization. The right legs of the same ticks were processed similarly, except that only 5 µl of 70% formic acid (FA) were initially added and the legs were homogenized using a plastic micro pestle (Carl Roth GmbH + Co. KG). Subsequently, an additional 15 µl of 70% FA were added (final volume 20 µl). The resulting homogenized solutions were purified using a hydrophilic MultiScreen PTFE filter plate with 0.45 µm pore size [Merck KGaA, Darmstadt, Germany] combined with a 96-well V-bottom collection plate and centrifuged at 2500 × *g* for 1 min in a plate centrifuge. Extracts were then spotted onto the MALDI target as described previously. The total incubation time in FA did not exceed 3 min.

The new sample preparation and protein extraction protocol was further compared to the protocols of Beltran et al. (2023) and Galletti et al. (2024). For each comparison, the left legs of three ticks were processed with the new protocol, and the right legs of the same ticks were processed according to the respective published protocol (new protocol vs. Galletti et al.; new protocol vs. Beltran et al.). For each protocol, the hands-on sample preparation time per target was recorded using a laboratory timer. Timing started when the tick stored in ethanol was retrieved from the microcentrifuge tube and ended after the last matrix spot on the target was completely dry. The reported preparation time represents the mean of three independent replicates.

During the comparison of the new protocol with that of Galletti et al. (2024), the same MALDI target spots generated from the leg extracts were also used to document matrix crystal morphology. Because the two protocols differ in spotting temperature (21 °C vs. 35 °C), representative spots prepared under each condition were photographed directly on the target after drying.

To evaluate the influence of different spectral acquisition methods on identical sample material, the same MALDI target spots from left-leg extracts prepared with the new workflow (three ticks) were measured twice. Each spot was first acquired using the standard acquisition method “MBT_AutoX_FilFungi_2” and then re-measured using the acquisition method “MBT_AutoX”. Spectra obtained with both acquisition methods from the same spots were subsequently compared.

#### MBT Biotarget 96 performance evaluation

Single use Bruker MBT Biotarget 96 [Bruker Daltonics GmbH & Co. KG, Bremen, Germany] were compared with the here used reusable Bruker MSP 96 polished steel BC target [Bruker Daltonics GmbH & Co. KG, Bremen, Germany]. Therefore, three MSPs of three male adult unfed *I. persulcatus* ticks (*n* = 3) were created with the here published method on steel targets. The same extracts from these ticks were spotted in addition on a Bruker MBT Biotarget 96 and in all other aspects treated as described above. This resulted in another three MSPs from the same ticks. The individual MSPs from the tick extracts on MBT Biotarget 96 were then tested against the MSPs derived from the same tick extracts on MSP 96 polished steel BC targets with Compass Explorer v. 4.1. Log(scores) were then averaged with the standard deviation indicated.

#### Identification

Twenty MSPs were created as described from adult female non engorged ticks [two *I. ricinus* (flagged), three *I. ricinus* (lab-colony), five *D. reticulatus* (flagged), five *H. dromedarii* (lab-colony) and five *I. persulcatus* (lab-colony)]. This is now described as pilot reference library. In addition, the preparation time for one target (eleven samples + controls) was measured beginning with retrieving the tick from the ethanol filled microcentrifuge tube, and the end point was the last dried matrix spot.

An additional twelve adult female non engorged ticks [two *I. ricinus* (flagged), one *I. ricinus* (lab-colony), three *D. reticulatus* (flagged), three *H. dromedarii* (lab-colony) and three *I. persulcatus* (lab-colony)] were extracted as described and spotted (one spot per tick). These spots were tested (only one single spectrum) and identified against the reference library resulting in identity log(scores). These log(scores) were calculated using MALDI Biotyper Compass Explorer v. 4.1 under standard settings from three ticks per species and were identified against the reference library. All identifications and comparisons via Compass Explorer v. 4.1 result in a log(score) from 0 to 3, where 0 indicates no similarity between the sample and the pilot reference library, and 3 indicates a 100% match. The score ranges used here for interpretation and colour-coding are the default categories defined by the manufacturer and were applied unchanged. The threshold for identifications is 1.699. Therefore, identification results below 1.7 are marked red. Log(scores) from 1.7 to 1.999 should provide a probable genus identification and are marked yellow. Log(scores) from 2 to 2.299 provide a secure genus and a probable species identification and log(scores) from 2.3 to 3 provide a highly probable species identification and are both marked in green. These categories were originally established for microbial identification (Wieser et al. 2012); in arthropod studies, thresholds of approximately 1.7–1.8 have commonly been applied for reliable identification (Yssouf et al. 2013; Boyer et al. 2019; Huynh et al. 2021). Retaining the unmodified default settings was a deliberate choice to keep the workflow directly transferable to routine diagnostic laboratories. Resulting log(scores) were averaged with the standard deviation being indicated. The underlying MSPs were created as described before.

#### Use of artificial intelligence

This article was prepared by the authors and improved with the help of large language models (LLMs).

All scientific content, including the study design, data analysis, interpretation and the initial drafting of all sections, was created by the authors. FHGenie (Fraunhofer in‑house LLM platform based on GPT‑5.1) was used to translate and to revise author‑written text and to obtain suggestions for language, style and grammar. No scientific content was generated de novo by AI tools. We confirm that no internal or sensitive research data and no personal data were uploaded to external LLM systems. For external tools (Perplexity Pro, DeepL), only publicly accessible search terms and text fragments were used for literature search and translation.

## Results

To enable a standardized, validated protocol that is efficient and feasible for routine hospital and laboratory settings, diverse tick specimens (e.g., different stages or species) were processed using a modified leg extraction procedure. Observations during method development showed, that ticks stored in 100% ethanol seemed to be more brittle than ticks stored in 70% ethanol, as legs from ticks stored in 70% ethanol were easier to remove and transfer into a reaction tube. Preliminary experimental approaches (Tables 1 and 2) demonstrated that many sample preparation approaches produced spectra. The cost–benefit ratio (efficiency) was evaluated by taking into account the materials and scope of work involved, as well as the resulting spectral quality (criteria: number of peaks, peak intensities, baseline, signal-to-noise ratio, peak distribution). In addition, the ease and cost-effectiveness of adapting the method for use in diagnostic laboratories were considered in the selection process.

**Table 2:** Results derived from preliminary approaches (Table 1) tested in the early stage for the establishment of a novel tick leg protein extraction method

Homogenization Methods		Result
	H1: Plastic micro pestle (tested with E1-3, SP1 and E1-4, SP2)	It should be observed if every single tick leg has been homogenized correctly. It might be possible, that some fragments remain intact and visible on some target spots. A drill machine can also generate plastic dust, which ends up in the extract, which might influence the results quality. Nevertheless, distinct peaks were observed
	H2: Ultrasonic bath (tested with H1, E1, E1a, E1b, SP1; E1, E1a, E1b, H6, SP3)	Incubating the homogenized or intact legs in an ultrasonic bath had no effect on the intensity or height of the resulting peaks compared to the controls
	H3: Pulverization with liquid Nitrogen (tested with E1, SP1)	This method achieved the finest homogenization of the legs. Homogenization quality was appreciated by visual observation and no leg fragments were seen. Distinct peaks were observed
	H4: Pulverization with dry ice (CO_2_) (tested with E1, SP1)	This method achieved the second finest homogenization of the legs. Distinct peaks were observed. Homogenization needed up to four cooling/vortexing cycles
	H5: Chitinase enzyme + FA extraction. (tested with H1, SP1, E1 and H6, SP3, E1)	Only a few distinct peaks were observed with a low signal to noise ratio
	**H6: No homogenization** **(just incubation)** (tested with E1, SP3; E1a-4, SP3;)	Distinct peaks were observed regardless of the incubation period. Incubation for 3 to 5 min (with E1) produced consistently intense spectra with no discernible difference between these durations. Extending incubation to 10 or 15 min yielded slightly higher intensities
Extraction Solutions	**E1: FA (70%)** E1a: FA (90%) E1b: FA (100%)	All extraction solutions worked in principle, and distinct peaks were produced
	E2: 50% Acetonitrile, 35% FA, 15% H_2_O (v/v)	Extraction solution worked in principle, and distinct peaks were produced
	E3: Acetonitrile (75%), H_2_O (25%), (v/v), followed by adding FA (75%), dest. H_2_O (25%), (v/v) separately. Same in reverse order was also tested	The extraction solutions worked in principle (also in reverse order), and distinct peaks were produced
	E4: 100% Acetone (−20 °C)/Standard protein precipitation protocol - pelletation, evaporation and reuptake in 75% FA as solvent	Extraction solution worked in principle, and distinct peaks were produced, but only in combination with homogenization
Sample Purification	SP1: PTFE Filter Membrane (hydrophilic) 0.2 µm or 0.45 µm	Prevented small leg fragments or plastic abrasion in the sample and on the target and distinct peaks were produced
	SP2: Centrifugation at 20,000xG for 3 min	Does not completely prevent small leg fragments or plastic abrasion in the sample and distinct peaks could be produced
	**SP3: none**	Further sample purification is not necessary as the tick legs remain intact and these will not fit into the pipette tip when removing the supernatant

Approaches were labeled by order and category as H1–6 for homogenization, E1–4 for extraction, and SP1–3 for sample purification. The precursors to the later method are printed in bold here

**Fig. 2:**
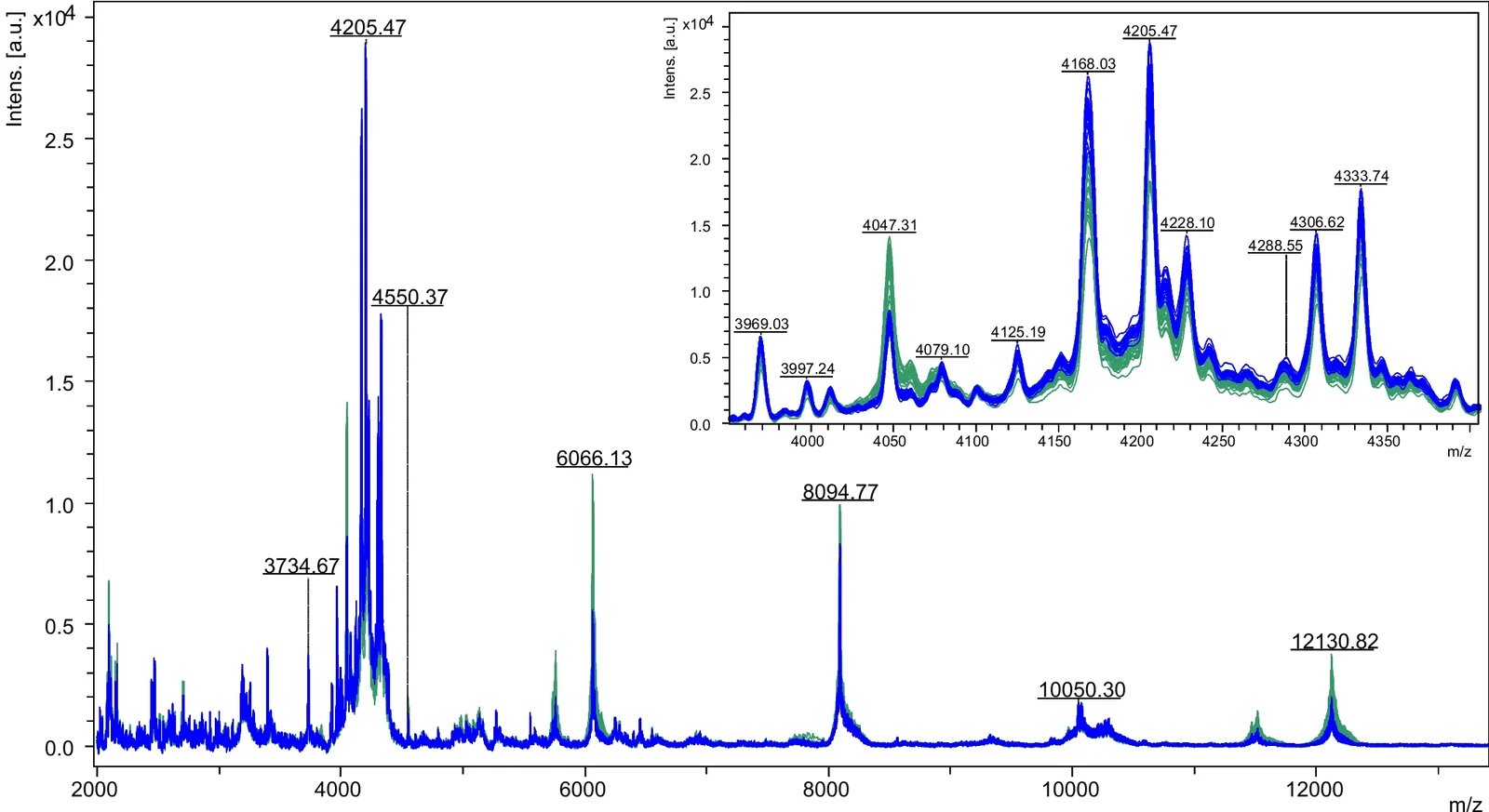
Reproducibility of MALDI-TOF MS spectra from the right (green) and left (blue) four legs of the same *Dermacentor reticulatus* tick. Tested was the reproducibility of the new workflow and comparability of the proteome in-between left and right four legs. Spectra from one tick are shown here. Almost all peak masses are present in both samples, while only the intensity from distinct peaks (e.g.: 4168 m/z (mass-to-charge ratio), 6066 m/z and 8094 m/z) differs, 4168 m/z is more intense in the left legs, 6066 m/z in the right legs

Reproducibility of the new workflow and comparability in-between the left four legs and the right four legs of the same tick was tested by visual inspection of three individual biological replicates of unfed female adult *D. reticulatus* (*n* = 3; left four legs vs. right four legs from three ticks) in Figs. 2, 9 and 10 as well as with creation and the mathematical similarity comparison of the MSPs of these spectra resulting in log(scores). Mean log(score) ± SD values (left four legs vs. right four legs from three ticks) were at 2.77 ± 0.07.

**Fig. 3:**
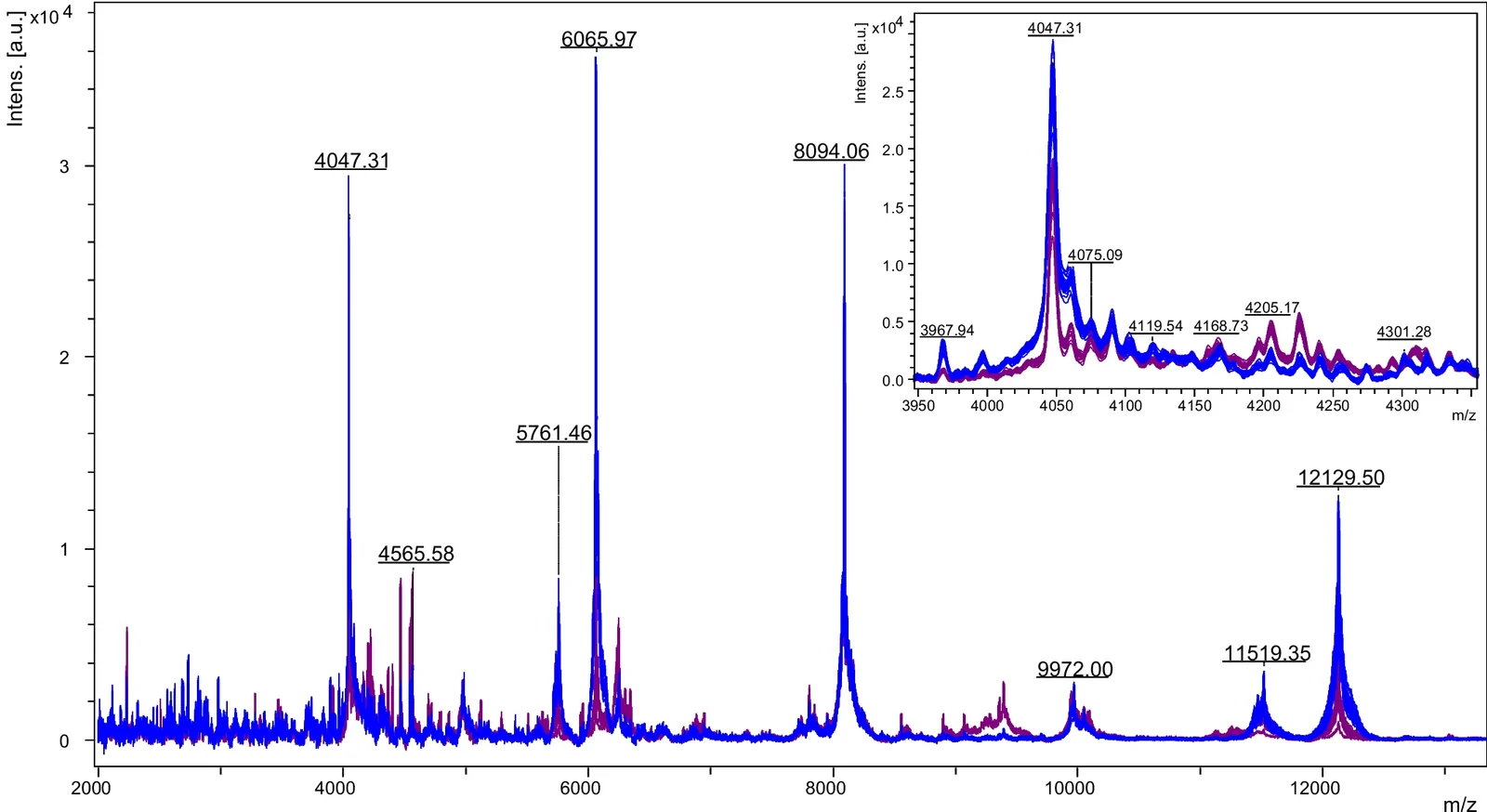
Comparison of the influence of homogenization on the MALDI-TOF MS spectra of a *D. reticulatus* tick. Left four legs (blue) were handled with the new workflow as stated in the methods section. Right four legs (purple) were additionally ground with a plastic micro pestle in the reaction tube

Homogenization with a micro pestle in comparison to incubation of whole legs led to some changes in the intensity of several peaks, but not to m/z shifts. This was tested by visual inspection of three individual biological replicates (*n* = 3) of adult unfed female *D. reticulatus* ticks (left four legs vs. homogenized right four legs from three ticks) in Figs. 3, 11, 12 and 13. Most intense peaks were lower with homogenization, and some lower ones were more intense.

**Fig. 4:**
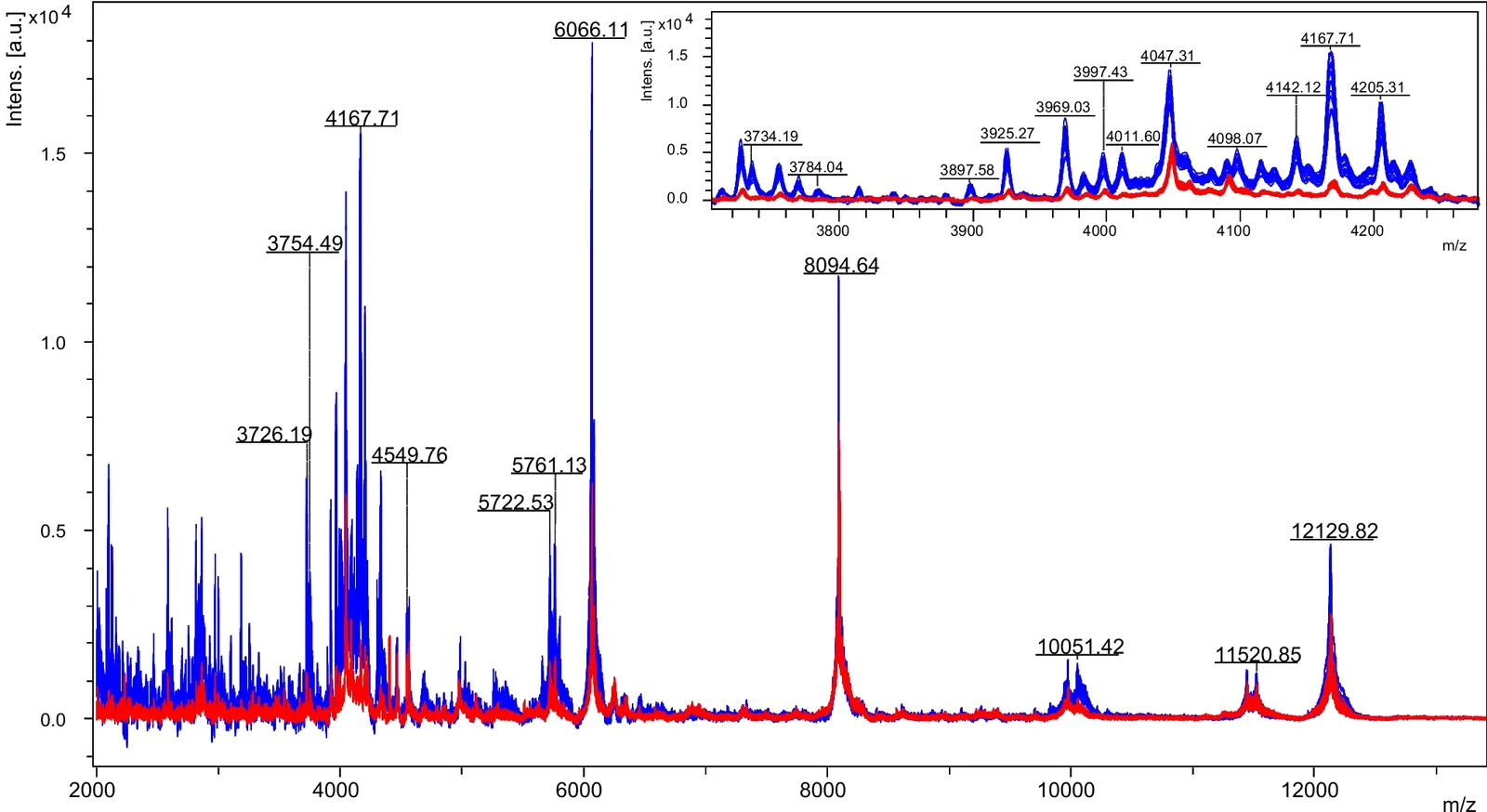
Comparison of the MALDI-TOF MS spectra of a *D. reticulatus* tick. Left legs were processed with the new workflow (blue) and right legs with the recently published procedure by Beltran et al. (2023) (red)

The new sample preparation and protein extraction protocol without homogenization was compared with recently published and functional methods from Beltran et al. (2023). This performance comparison was evaluated by visual inspection of three individual biological replicates of adult unfed female *D. reticulatus* ticks (*n* = 3) (left four legs vs. right four legs from three ticks) in Figs. 4, 13 and 14. Major differences of the Beltran et al. (2023) protocol to the new workflow are: extraction solvent is 100% FA, homogenization with a micropestle applied in combination with centrifugation, 45 °C is used as spotting temperature, MBT_AutoX is used as acquisition method and no recalibration was used. The red spectrum (Fig. 4) is in general lower in abundance and some peaks are missing. The time required to extract the samples was comparable, at approximately 9 min for both protocols, provided that the time for a single sample was measured. The method published here allows for staggered processing, which was not possible with the Beltran et al. (2023) method, enabling two samples to be extracted and spotted in about 12:30 min. and eleven samples and controls (a whole full target) in about 60 min.

**Fig. 5:**
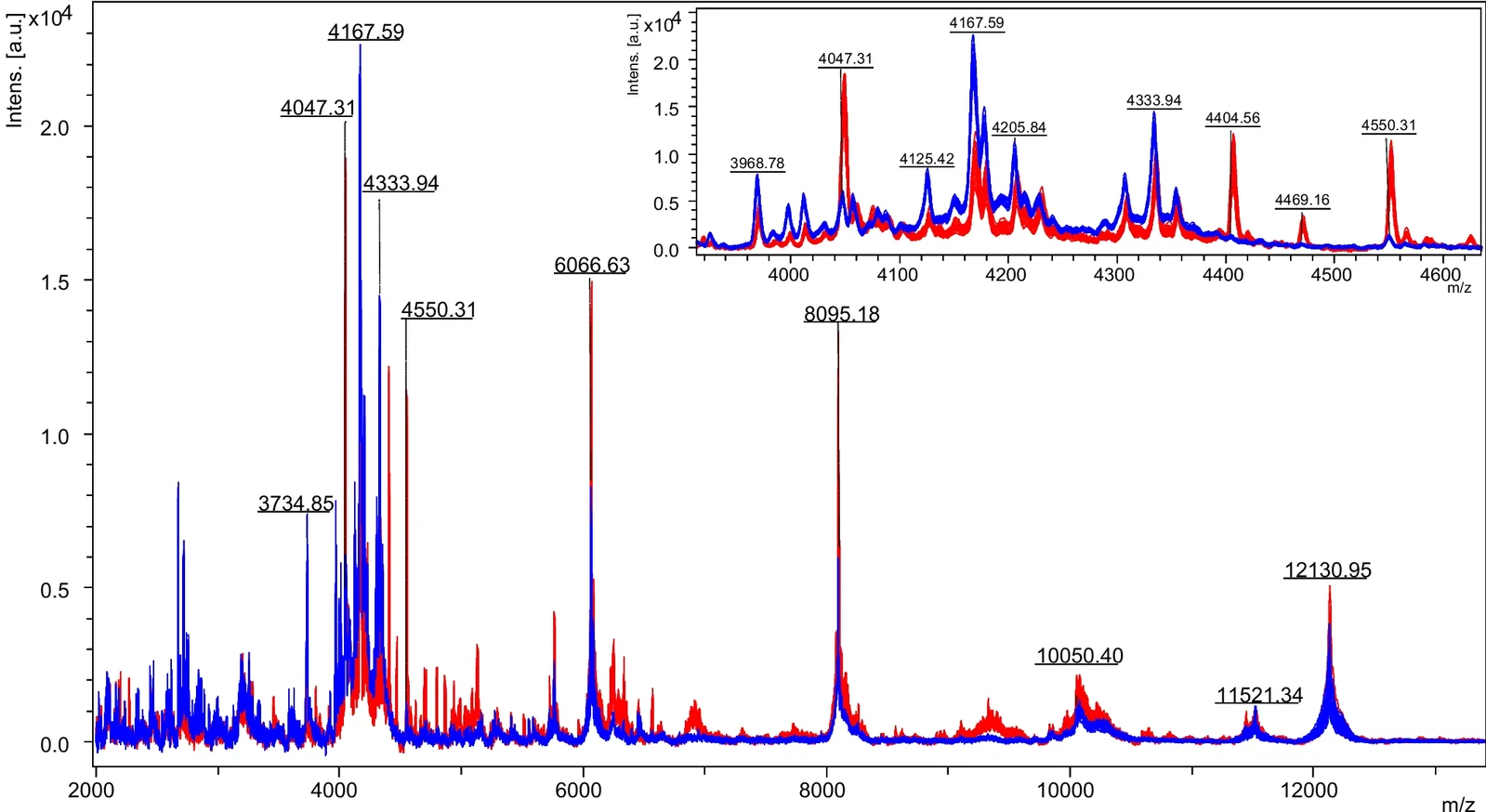
Comparison of the MALDI-TOF MS spectra of a *D. reticulatus* tick. Left legs were processed with the new workflow (blue) and right legs with the recently published procedure by Galletti et al. (2024) (red)

The comparison of the new sample protocol with the recently published and functional methods from Galletti et al. (2024) was evaluated by visual inspection of three individual biological replicates of adult unfed female *D. reticulatus* ticks (*n* = 3) (left four legs vs. right four legs from three ticks) in Figs. 5, 15 and 16. Major differences of the Galletti et al. (2024) protocol to the new workflow are: air dry (2 h) after leg dissection, legs were washed with acetonitrile, extraction solvent was 20 µl of each of 70% FA & 100% acetonitrile (v/v), homogenization with a ball mill and 5-mm steel ball and centrifugation. Room temperature was used as spotting temperature, MBT_AutoX was used as acquisition method and no recalibration was used. The red spectra (Fig. 5), which were produced according to the method of Galletti et al. (2024), in Figs. 5, 15 and 16, show mostly slightly more intense peaks compared to the new workflow (blue). The time required to extract the samples was about 2 h and 15 min for the procedure by Galletti et al. (2024) and about 9 min for the here published protocol.

After optimization of the extraction, the subsequent step in sample preparation was target spotting. Spotting at 35 °C produced more homogeneous, uniformly distributed, smaller matrix crystals, whereas room temperature spotting (21 °C) yielded larger, discrete crystals.

Further single-use Bruker MBT Biotarget 96 plates were compared with Bruker MSP 96 polished steel BC targets by spotting same extracts of three male, unfed, adult *I. persulcatus* ticks on both targets, resulting in two MSPs per tick. Spectra acquired from the same extracts on both target types were virtually indistinguishable, with no additional or missing peaks and no mass shifts (Figs. 19, 20 and 21); the two MSPs from three ticks tested against each other yielded a mean log(score) of 2.86 ± 0.03.

The comparison between the same tick sample spots measured with the Bruker acquisition methods MBT_AutoX and MBT_AutoX_FilFungi_2 was evaluated by visual inspection of three individual biological replicates (*n* = 3) (spots derived from four legs with MBT_AutoX vs. same spots with MBT_AutoX_FilFungi_2 from three adult unfed female *D. reticulatus* ticks) in Figs. 7, 17 and 18. In direct comparison, the spectra measured with the MBT_AutoX_FilFungi_2 (blue) method are slightly more intense but show a reduced variance in intensity (all spectra within a set of spectra show more similar intensities).

Figure 8 shows single spectra from individuals of different tick species and life stages, extracted and processed with the new workflow as proof of principle for the applicability of the developed method.

We evaluated identification performance using a pilot MALDI-TOF MS reference library comprising five MSPs per species for four tick species (*I. ricinus*,* D. reticulatus*,* H. dromedarii*, and *I. persulcatus*; *n* = 20). All ticks were female, adult and unfed. An independent test set of three ticks per species (*n* = 12) was queried against this library. All 12/12 tick spectra were correctly assigned at the species level, concordant with the reference morphological/molecular identifications, with no misidentifications. Mean log(scores) ± SD by species were:

*I. ricinus* 2.31 ± 0.11, *D. reticulatus* 2.49 ± 0.04, *H. dromedarii* 2.66 ± 0.02 and *I. persulcatus* 2.40 ± 0.07. The highest (log) score which assigned a wrong species (*I. ricinus*) in this experiment was 1.56 as fourth rank marked in red (not reliable identification), derived by one *I. persulcatus* tick (rank one to three were assigned correctly).

All ticks were identified morphologically, and five specimens per species were randomly selected for molecular confirmation. The resulting sequences (accession numbers) for *I. ricinus* PZ811716 - PZ811720), *D. reticulatus* (PZ811701 - PZ811705), *H. dromedarii* (PZ811706 - PZ811710) and *I. persulcatus* (PZ811711 - PZ811715) shared ≥ 99.12% identity and 100% coverage with their closest GenBank match and confirmed the morphological identification in all cases. Since several sequences of the same species returned an identical top BLAST hit, only one or two reference accession numbers are listed per species: *I. ricinus* (PV113054.1, OY973613.1), *D. reticulatus* (OR936113.1), *H. dromedarii* (ON937757.1, LC775364.1) and *I. persulcatus* (MH790201.1).

## Discussion

This study compared different published and unpublished MALDI-TOF MS extraction methods (Tables 1, 2 and 3, Figs. 3, 4, 5, 11, 12, 13, 14, 15 and 16), spotting techniques (Fig. 6), acquisitions varieties (Figs. 7, 17 and 18) and data processing methods (Fig. 1) to identify and develop the most promising approach to setup the base for the establishment of a novel tick identification method aiming for optimized and reproducible MALDI-TOF MS spectra.

**Table 3:** Discussion of preliminary approaches (Tables 1 and 2) tested in the early stage for the establishment of a novel tick leg protein extraction method

Homogenization Methods		Discussion
	H1: Plastic micro pestle (tested with E1-3, SP1 and E1-4, SP2)	This approach is unsuitable for diagnostic purposes, as reproducibility cannot always be guaranteed; it is sometimes difficult to grind all the legs evenly. It only seems suitable when it has been ensured that all legs have been crushed evenly, but in principle, sufficiently intense peaks are produced. Post-Filtration with a PTFE Filter Membrane (0.2–0.45 µm) is highly recommended to ensure that no fragments are still in the sample which can cause disturbances in the MALDI-TOF MS
	H2: Ultrasonic bath (tested with H1, E1, E1a, E1b, SP1; E1, E1a, E1b, H6, SP3)	The expected improvement in extraction efficiency did not occur, as no change in the spectra could be detected. The method is either unsuitable for tick legs, or the maximum extraction efficiency has already been reached
	H3: Pulverization with liquid Nitrogen (tested with E1, SP1)	Due to the higher work load for sample preparation and the need for liquid nitrogen (hazardous substance), this method is unsuitable for the objectives of this study
	H4: Pulverization with dry ice (CO_2_) (tested with E1, SP1)	Due to the higher work load for sample preparation and the need for dry ice (expensive and hazardous substance), this method is unsuitable for the objectives of this study
	H5: Chitinase enzyme + FA extraction. (tested with H1, SP1, E1 and H6, SP3, E1)	Seems not suitable due to a low signal to noise ratio
	**H6: No homogenization** **(just incubation)** (tested with E1, SP3; E1a-4, SP3;)	**Highly efficient sample preparation method. A 3 min incubation provided the best balance, with a favorable cost-to-benefit ratio – especially combined with other optimizations**
Extraction Solutions	**E1: FA (70%)** E1a: FA (90%) E1b: FA (100%)	**FA (70%) was chosen because of its effectiveness for extraction of tick legs and its widespread availability given to the widespread use of 70% FA in routine microbial diagnostics. Any more complex method was ruled out for this diagnostic purpose**
	E2: 50% acetonitrile, 35% FA, 15% H_2_O (v/v)	See E1
	E3: Acetonitrile (75%), H_2_O (25%), (v/v), followed by adding FA (75%), dest. H_2_O (25%), (v/v) separately. Same in reverse order was also tested	See E1
	E4: 100% Acetone (−20 °C)/Standard protein precipitation protocol - pelletation, evaporation and reuptake in 75% FA as solvent	See E1
Sample Purification	SP1: PTFE Filter Membrane (hydrophilic) 0.2 µm or 0.45 µm	Both are very appropriate for preventing small leg fragments or plastic abrasion in the sample, which may cause inconstancies or even damage to the MALDI-TOF MS. A filter membrane was not necessary for the new method since the legs are staying intact and simple pipetting prevents material transfer
	SP2: Centrifugation at 20,000xG for 3 min	In combination with homogenization, it did not completely prevent small fragments from remaining in the solution, due to the buoyancy of some of the fragments, which is why centrifugation was deemed insufficient. Centrifugation was not necessary for the new method since the legs are staying intact and simple pipetting prevents material transfer
	**SP3: none**	**Highly efficient and therefore selected**

**Fig. 6:**
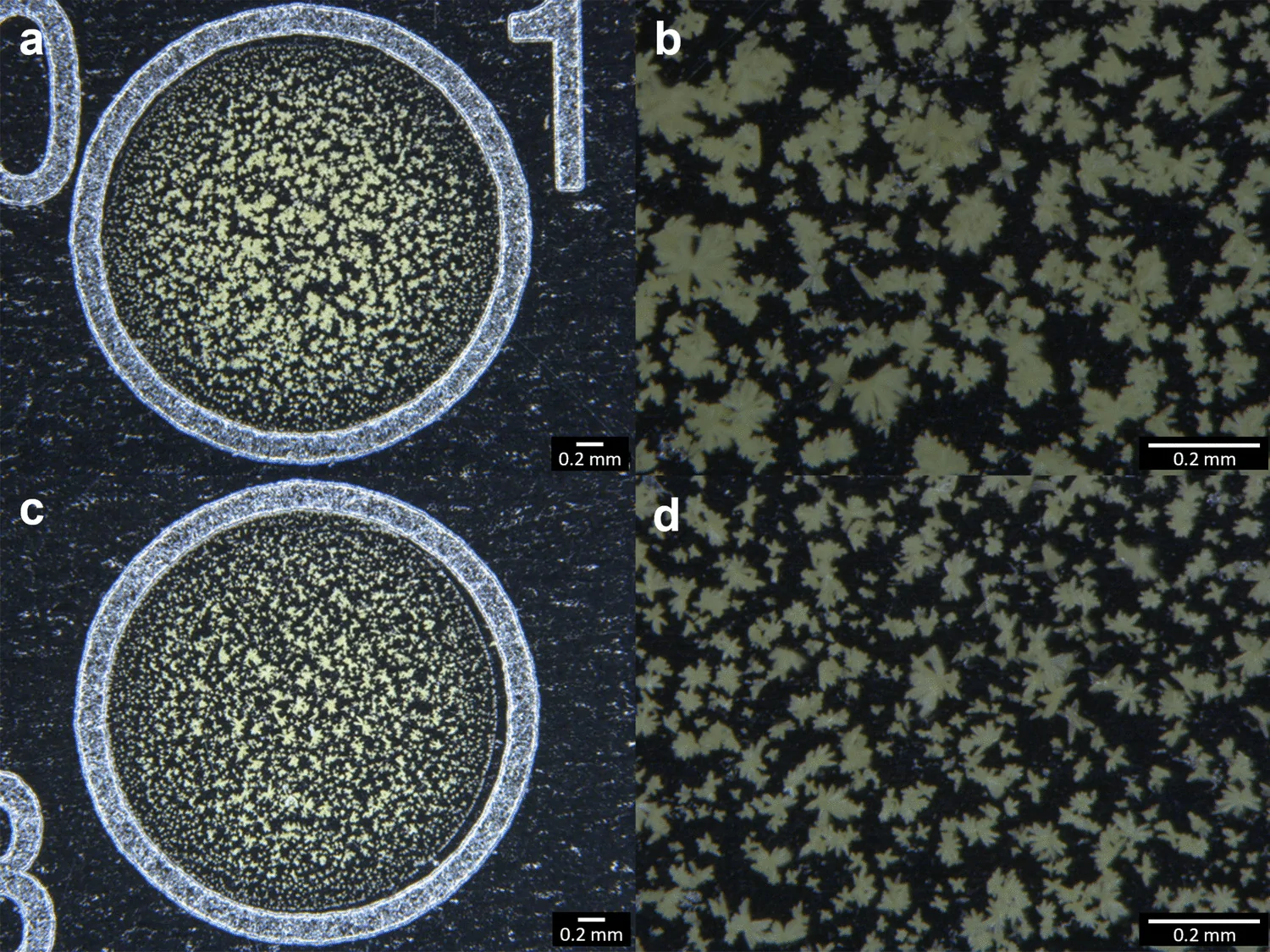
Bruker MSP 96 polished steel BC target with sample spots. a, b: spotting at room temperature (21 °C); c, d: spotting at 35 °C. Scalebar represents 0.2 mm

**Fig. 7:**
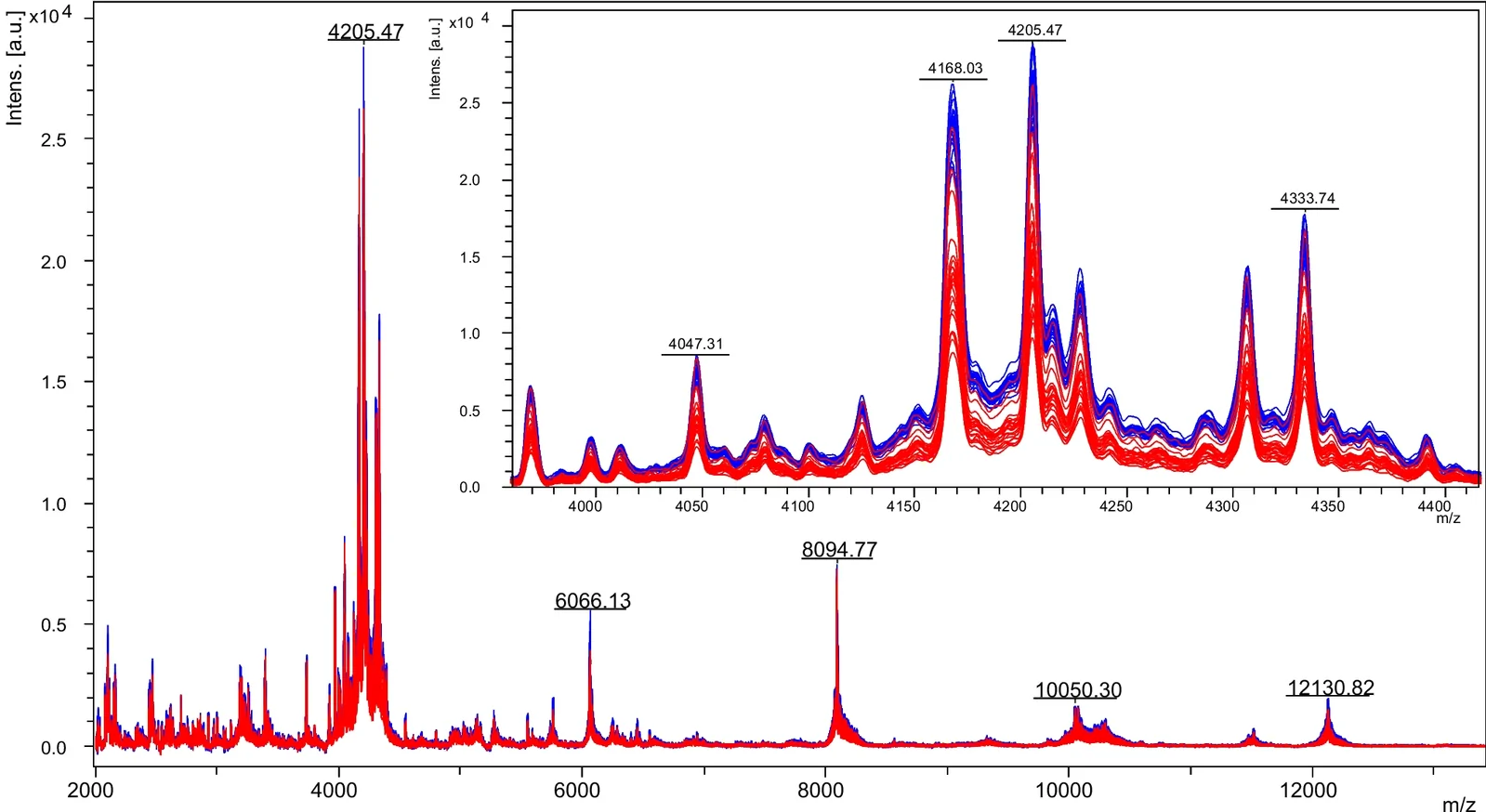
Comparison of the Bruker acquisition methods MBT_AutoX and MBT_AutoX_FilFungi_2 with tick samples. The exact same eight spots of a *D. reticulatus* tick sample was measured with the MBT_AutoX (red) and MBT_AutoX_FilFungi_2 method (blue)

The storage of tick samples is the first factor, which could influence the preparation and molecular preservation (Marquina et al. 2021). Depending on the research question, tick samples might be tested fresh, dried, frozen (at −20 °C or −80 °C) or after storing in 70% ethanol p.a. (Chitimia-Dobler et al. 2019; Król et al. 2020, 2024; Marquina et al. 2021; Wimbauer et al. 2022). Ethanol appears to be the most widely used solution for preserving ticks, as it is readily available, preserves insects very well over a long period of time, and is easy to use, especially in field conditions (Marquina et al. 2021; Ahamada M'madi et al. 2022; Galletti et al. 2024). In addition, some species (ixodid ticks) require a storage period in ethanol for pathogen inactivation before they can be handled safely (Schulz et al. 2021). When using a high ethanol concentration (100%), brittleness can be quite challenging for the tick leg preparation. Storage in high ethanol concentrations is known to induce brittleness in insect samples (Marquina et al. 2021). For ticks, statistically robust evidence for identical effects across taxa are lacking, but qualitative observations during method development indicated that ticks stored in 100% ethanol were also more brittle compared to ticks stored in 70% ethanol. For MALDI‑TOF MS in particular, freezing at - 20 °C or - 80 °C has been reported to preserve protein profiles best (Nebbak et al. 2017). Where freezing is not feasible – as is usually the case for field‑collected or submitted specimens – 70% ethanol (v/v) is recommended for satisfactory handling, storage, preparation and molecular preservation for a storage time of at least up to six months (Nebbak et al. 2017; Marquina et al. 2021). For the extraction, all four legs of one side of the ticks were used. At first, several extraction approaches were pretested for scientific and economic performance (Tables 1, 2 and 3). Feasibility for diagnostic implementation was defined as compatibility with standard equipment and reagents already established in routine diagnostic laboratories, avoiding specialized devices (e.g., ball mills) and any additional new chemicals or instruments to avoid additional expenses.

Approaches were labeled by order and category as H1–6 for homogenization, E1–4 for extraction, and SP1–3 for sample purification. The precursors to the later method are printed in bold here.

It turned out that homogenization of the tick legs was not necessary to obtain high intense spectra, which was tested and demonstrated in Figs. 3, 11 and 12 as well as the importance of standardized sample handling. The reproducibility and comparability of the spectra between the left and right four legs were tested and verified in Figs. 2, 9 and 10. The contrasting intensity differences of individual peaks (e.g., 6066 m/z or 8094 m/z) cannot be clearly explained and may be caused by metabolic differences or, more likely, by ionization efficiency differences. The mass-to-charge ratio (m/z) of multiple charged ions is composed for single charged (z = 1): m/z = M + H or for double charged (z = 2): m/z = (M + 2H)/2. Thus, for example, 8094 m/z may correspond to the single charge and 4047 m/z to the double charge of the same molecule**.** Different multiple charged ion distributions should be considered when comparing different samples by just looking on different spectra. However, this special kind of intensity differences are normal in MALDI-TOF analysis and according to the similarity analysis via Compass Explorer v. 4.1 between left and right legs (which were extracted separately) of three ticks (n = 3), the spectra of the left and right legs are nearly identical with a mean log(scores) ± SD of 2.77 ± 0.07. This demonstrates the high reproducibility of the here published method to generate MSPs for tick identification.

To put the simplified new workflow to the test, we compared it with recently published and functional methods from Beltran et al. (2023) and Galletti et al. (2024). The extraction by Beltran et al. (2023) (Figs. 4, 13 and 14) works as they demonstrated. A good economic extraction method needs to be quick, valid, but also highly reproducible. Grinding with a micropestle is a legitimate method to use for several reasons, but centrifugation alone seems insufficient to completely avoid small tick fragments on the MALDI-TOF MS target, which might have negative consequences to reproducibility and in the worst case also to the device. This might be avoided by using PTFE filter membranes (0.20 or 0.45 µm) which ensure always a clear sample while experiencing a small volume loss of about 30% in the membrane. The slight mass shift between the peaks of the spectra derived from the two extraction protocols can be explained by the recalibration of the spectra obtained through the new method (blue) (Fig. 4). The extraction solvent used by Beltran et al. (2023) is 100% FA. In an aqueous system, FA acts as a proton donor towards water as the accepting base (Brønsted 1923; Lowry 1923). In neat FA, however, no external base is available; protonation depends on autoproteolysis, the pH concept is no longer applicable, and protonation conditions for the analytes are consequently less well defined. A defined aqueous fraction, as used here (70% FA, v/v), therefore provides more controlled and reproducible protonation conditions. Further, Beltran et al. (2023) recommend a spotting temperature of 45 °C. According to the manufacturer, the HCCA-Matrix needs a minimum drying time for optimal crystal growth. If the matrix dries and crystallizes too quickly, due to a higher temperature than 35 °C, smaller crystals may appear and could overlay each other. In the here published conditions, crystals will grow reproducible and sized, as intended by Bruker, attached to the target (Idelevich et al. 2023). We recommend 35 °C as well as the manufacturer as preferred spotting temperature (Fig. 6). From the dissection of the legs to the dried matrix, both methods take approximately nine minutes per tick, which means that there is no considerable advantage or disadvantage if only one tick is to be prepared. If two or more ticks have to be prepared in parallel, the new workflow allows the next ticks to be prepared or spotted during the incubation period of the first ones. This is more difficult with the Beltran et al. (2023) method, due to the grinding and centrifugation steps. Our method therefore also offers a substantial time (economic) advantage here if more than one tick should be prepared. For two ticks, the new method took about 12:30 min and eleven samples and controls (a whole full target) could be completed in about 60 min observing all the times specified in the methods part. In all cases, eight spots were applied per sample (MSP preparation). For identification purposes alone, a single spot may be sufficient, which should further reduce the time required and might enable sizable higher throughput compared to all other mentioned methods for tick identification in the future. The workflow of Galletti et al. (2024) appears to be fundamentally quite similar to the methods of Diarra et al. (2017), Boyer et al. (2019) and Huynh et al. (2021); these were therefore not tested separately. However, these are not the methods of choice for a routine identification of ticks, due to the considerable high time required (approx. 2 h and 15 min), the use of a ball mill, and the need for non-standard extraction solutions. As with Beltran et al. (2023), tiny leg particles on the target are almost unavoidable with this method. The purity of the isolates might also be further improved by using PTFE filter membranes. An improved spectrum quality would justify such additional effort, but the spectrum quality was not elevated compared to the new protocol.

Protocols based on mechanical homogenization of tick legs, whether by manual grinding or by abrasive/bead‑assisted disruption in a TissueLyser or ball mill (Diarra et al. 2017; Nebbak et al. 2017; Boyer et al. 2019; Galletti et al. 2024), differ fundamentally from the present approach as the leg tissue is crushed in all of these methods prior to extraction. In the protocol described here, proteins are extracted from the macroscopically intact legs, which remain in the reaction tube and is separated from the liquid phase by simple pipetting, without any grinding step, centrifugation, filtration, additional consumables or dedicated homogenization equipment.

As already briefly mentioned previously with the protocol by Beltran et al. (2023), the authors of this study also highly recommend an increased spotting temperature. Idelevich et al. (2023) reported improved identification rates and log(scores) for unknown bacterial samples when a 35 °C heating device instead of room temperature was used during target spotting; the study also noted time- and cost-saving benefits due to an approximately 50% reduction in drying time. Beltran et al. (2023) reported independently, that spotting at elevated temperature (45 °C) yields more homogeneous, uniformly distributed matrix crystals, a finding corroborated in the present study in Fig. 6. The manufacturer (Bruker) recommends 35 °C as the optimal spotting temperature to minimize heat-induced damage while preserving the benefits of homogeneous matrix crystallization.

Almost no difference could be detected in spectra between single-use Bruker MBT Biotargets 96 and the Bruker MSP 96 polished steel BC targets, as MSPs of identical tick isolates measured on both targets and compared using Compass Explorer v. 4.1 yielded a high mean log(score) of 2.86 ± 0.03.

Nevertheless, the decision was made to use steel targets for this study, as they have proven to be reliable, extremely durable, and more cost-effective in our laboratory setup. So, using different targets for the creation of reference entries and for the later identification might likely not have a negative impact on future identification log(scores). For automated mass spectral acquisition, the Bruker Microflex MALDI‑TOF MS was operated using flexControl Software v. 3.4 with the standard AutoXecute method MBT_AutoX and the modified method MBT_AutoX_FilFungi_2, originally developed for filamentous fungi (Fig. 7). The Biotyper standard spectra acquisition method MBT_AutoX employed the spiral small raster, acquiring 240 laser shots per spectrum in linear mode. In contrast, MBT_AutoX_FilFungi_2 accumulated up to 800 laser shots per spectrum and uses a smaller intensity threshold to ensure the successful acquisition at substantially lower initial signal intensities (e.g., after suboptimal sample processing or the use of suboptimal samples). If the quality or intensity of the spectra was already good, the MBT_AutoX_FilFungi_2 additionally used an early‑termination algorithm that halted acquisition once predefined quality thresholds were reached. This configuration (MBT_AutoX_FilFungi_2) produced spectra with reduced intensity variance along the y‑axis and improved inter‑replicate homogeneity which is beneficial for MSP creation. MBT_AutoX_FilFungi_2 was originally applied to samples expected to yield weak signals, such as filamentous fungi and is in the present study used for tick samples, where extended averaging improved the reliability of peak detection and will improve subsequent identification.

To optimize future identification of unknown tick samples, spectra were recalibrated to improve mass accuracy (see Fig. 1). This is also an enhancement in spectra quality and especially in comparability. Recalibration of the reference spectra is warranted because it corrects systematic mass offsets and thereby improves identification accuracy. For example, if the true mass is 6500 m/z and the unknown spectrum is incidentally shifted by 1500 ppm to a lower m/z ratio (≈ 6490 m/z), the peak remains within a ± 2000 ppm tolerance and is still matched. If the MSP is simultaneously miscalibrated by 1500 ppm to a higher m/z ratio (≈ 6510 m/z), the offsets sum, yielding an effective discrepancy of ≈ 3000 ppm (≈ 20 m/z), effectively exceeding the tolerance. Recalibration aligns the MSP to the true mass, limits cumulative errors, and increases the likelihood of correct matches across measurement runs (See Fig. 1).

**Fig. 8:**
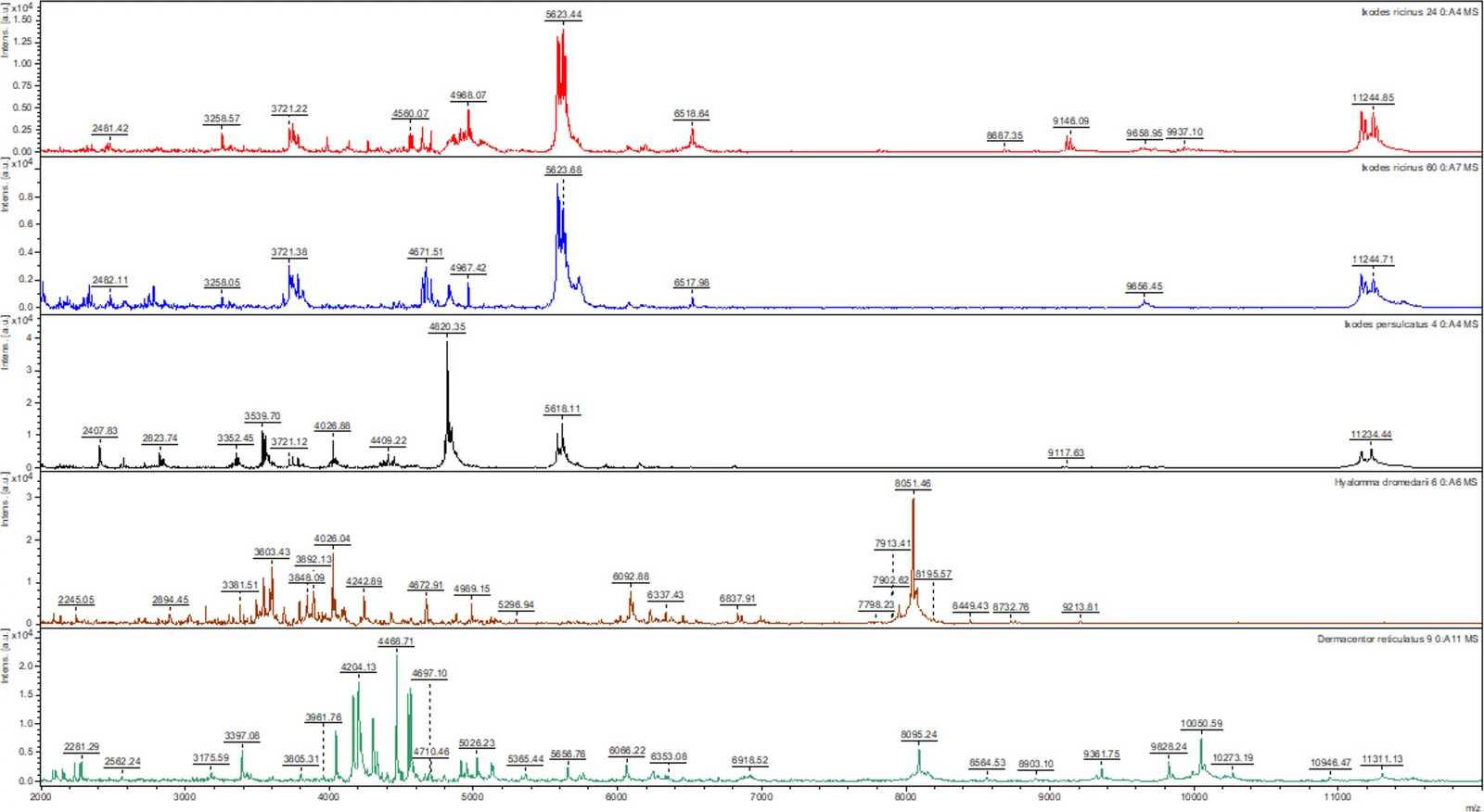
Recalibrated MALDI-TOF MS spectra of different tick species and stages, extracted and processed with the new workflow. *I. ricinus* (red: female, adult, unfed, lab colony), *I. ricinus* (blue: nymph, unfed, flagged, stored in 70% ethanol), *I. persulcatus* (black: female, adult, unfed, lab colony), *H. dromedarii* (brown: female, adult, unfed, lab colony), and *D. reticulatus* (green: male, adult, unfed, flagged)

The developed method was tested using several tick species in preliminary non-systematic tests, suggesting that it should work with other tick species as Fig. 8 and the pilot identification approach demonstrate. It is quicker, cheaper, easier for labs to adapt and gives at least the same quality in comparison to published methods. Besides, it may be easier to compare between labs – because of the easier protocol with less steps to involve personnel-related handling differences to potentially cause variation in results. Data from Fig. 8 and the pilot identification approach indicate that the newly developed extraction protocol generates MALDI-TOF MS spectra of sufficient quality for reliable species-level identification of ticks. Using a small pilot reference library (n = 20, including four species with five specimens), twelve out of twelve additional ticks (consisting of four species with three specimens) were correctly identified (100% identification rate), with high log(scores) (≥ 2.2) consistent with our species-level criteria (morphological and molecular identifications), supporting the suitability of the protocol for tick species level identification. Even closely related species *I. ricinus* and *I. persulcatus* were identified 100% correctly. However, these findings are preliminary. The evaluation used a small library, only adult unfed females from four species, and spectra produced under closely matched laboratory conditions, which may inflate apparent accuracy. Future work should assess robustness across additional species and near-neighbors, different life stages, nutritional/engorgement status geographic origins, operators and instruments and storage conditions and durations.

**Fig. 9:**
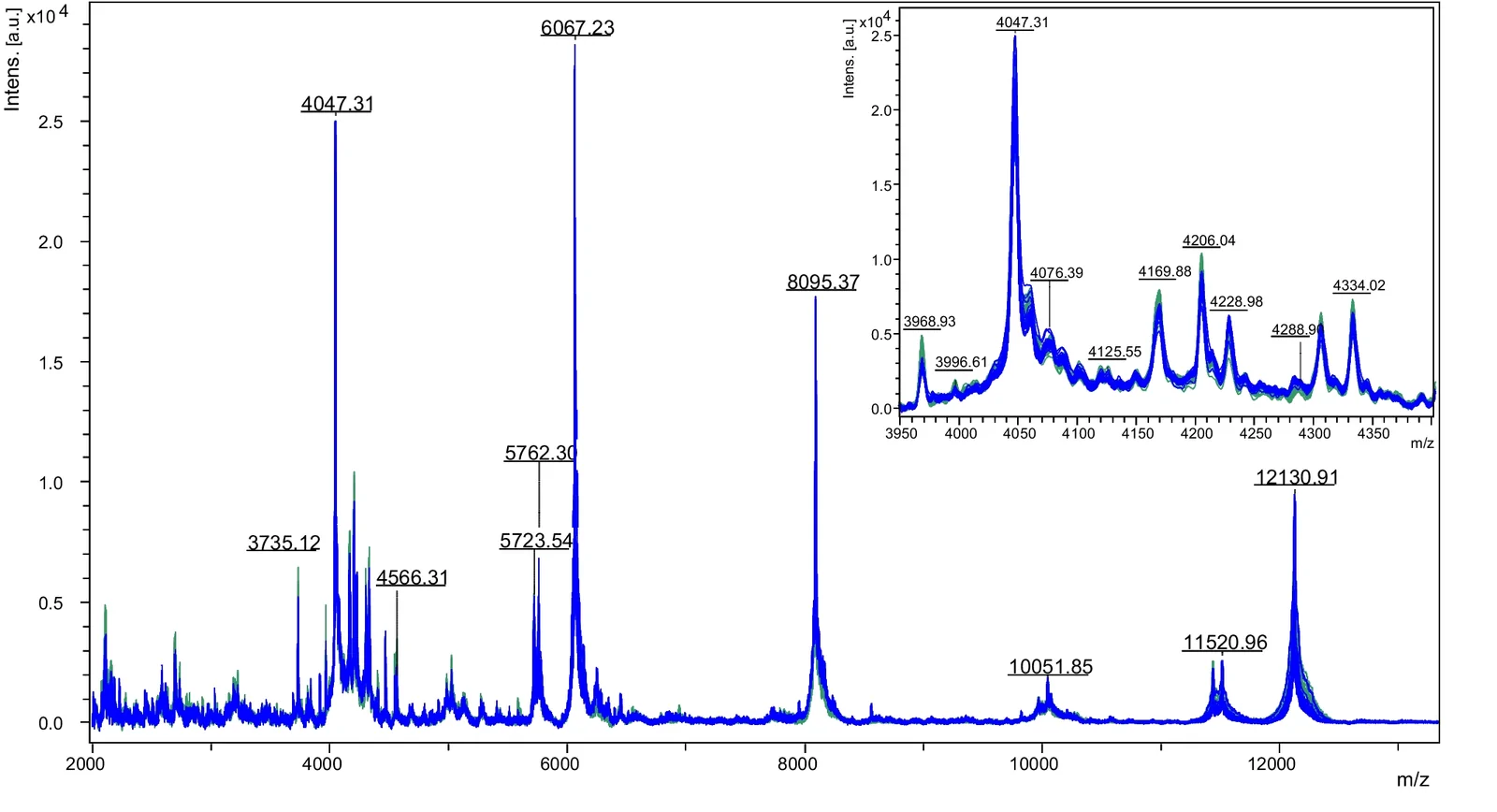
Reproducibility of MALDI-TOF MS spectra from the right (green) and left (blue) four legs of the same *D. reticulatus* tick (second replicate). Tested was the reproducibility of the new workflow and comparability of the proteome in-between left and right four legs. Spectra from one tick are shown here. All peak masses are present in both samples

**Fig. 10:**
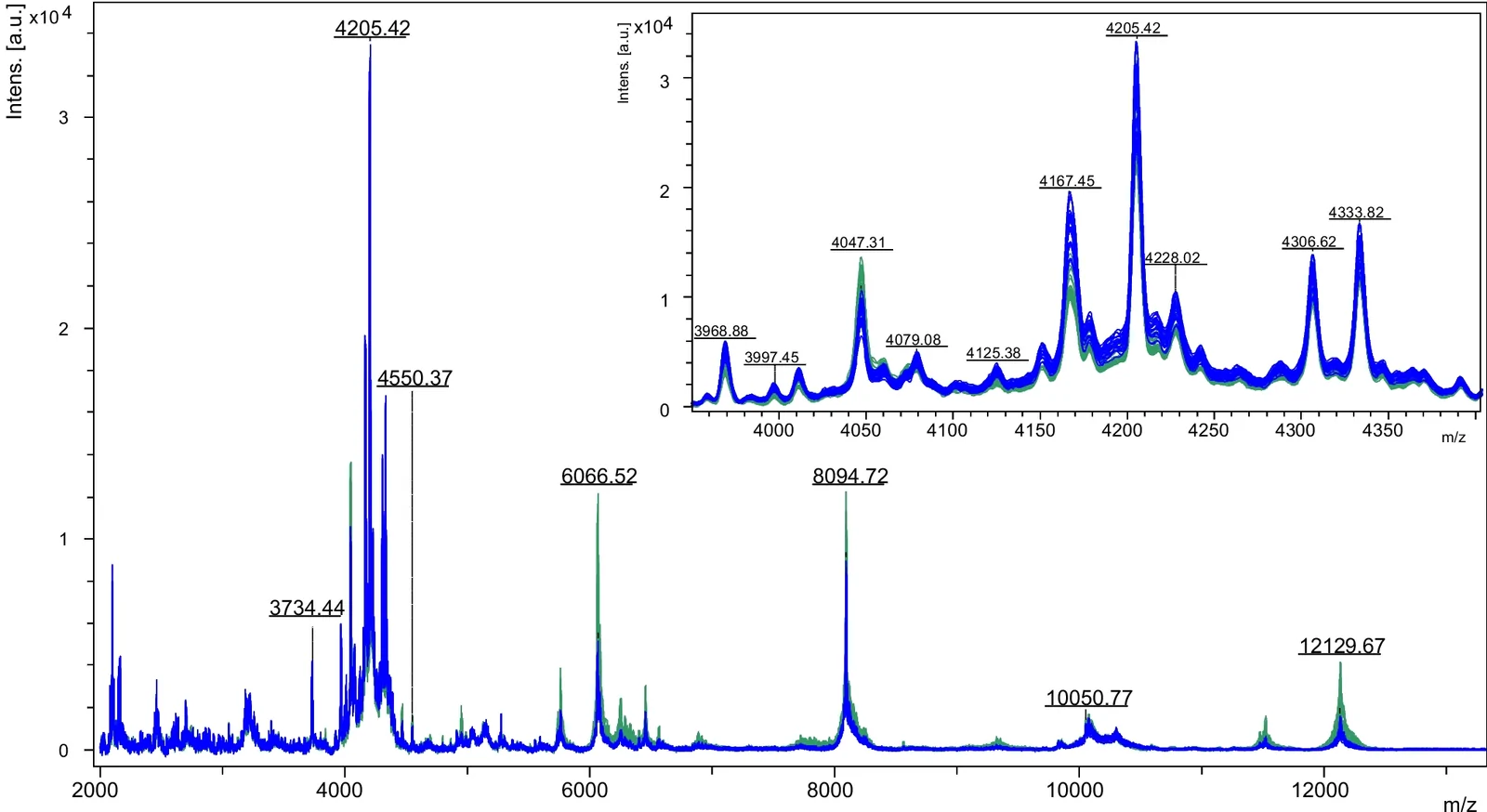
Reproducibility of MALDI-TOF MS spectra from the right (green) and left (blue) four legs of the same *D. reticulatus* tick (third replicate). Tested was the reproducibility of the new workflow and comparability of the proteome in-between left and right four legs. Spectra from one tick are shown here. All peak masses are present in both samples

**Fig. 11:**
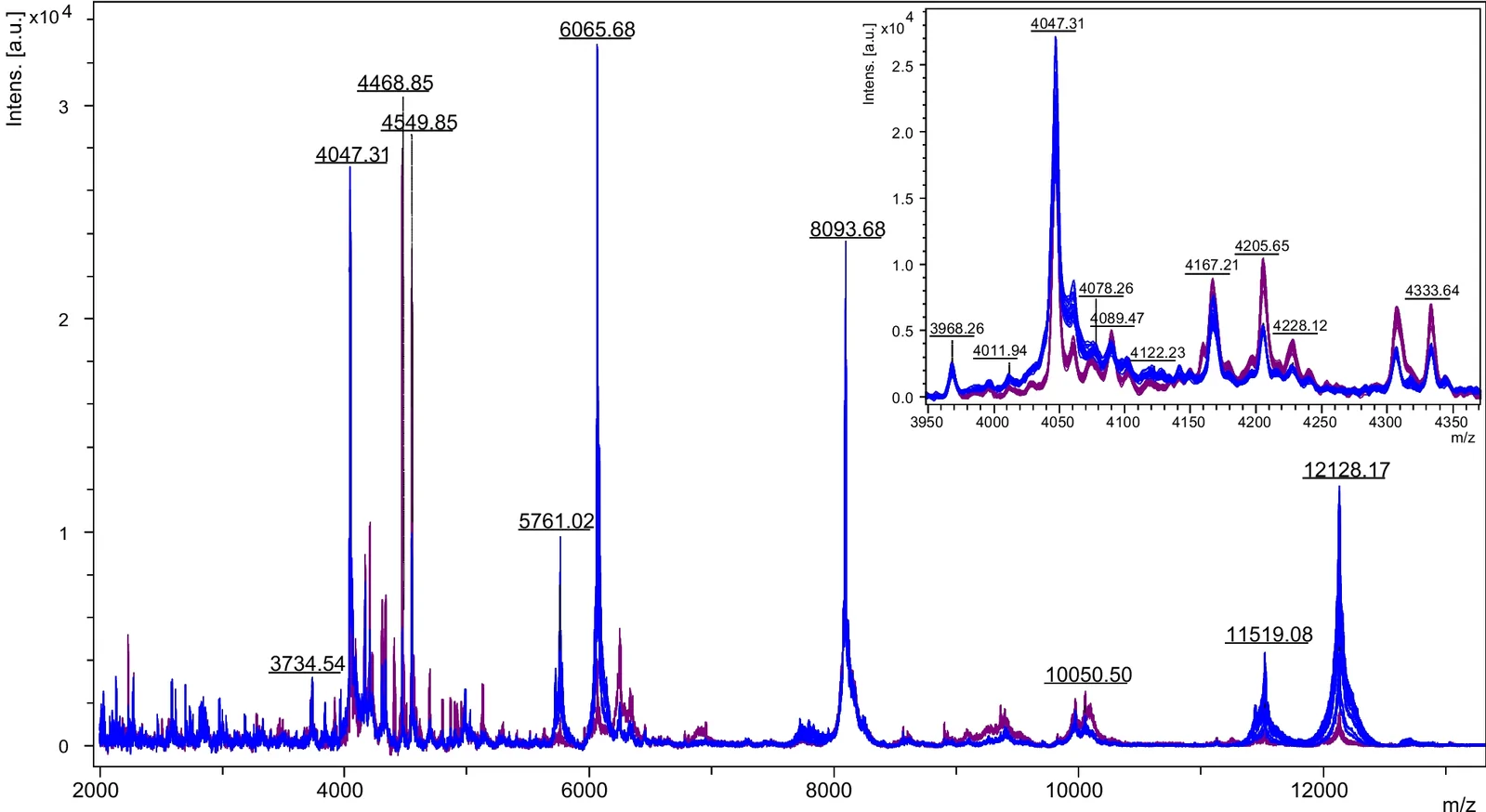
Comparison of the influence of homogenization on the MALDI-TOF MS spectra of a *D. reticulatus* tick (second replicate). Left four legs (blue) were handled with the new workflow as stated in the methods section. Right four legs (purple) were additionally ground with a plastic micropistill in the reaction tube

**Fig. 12:**
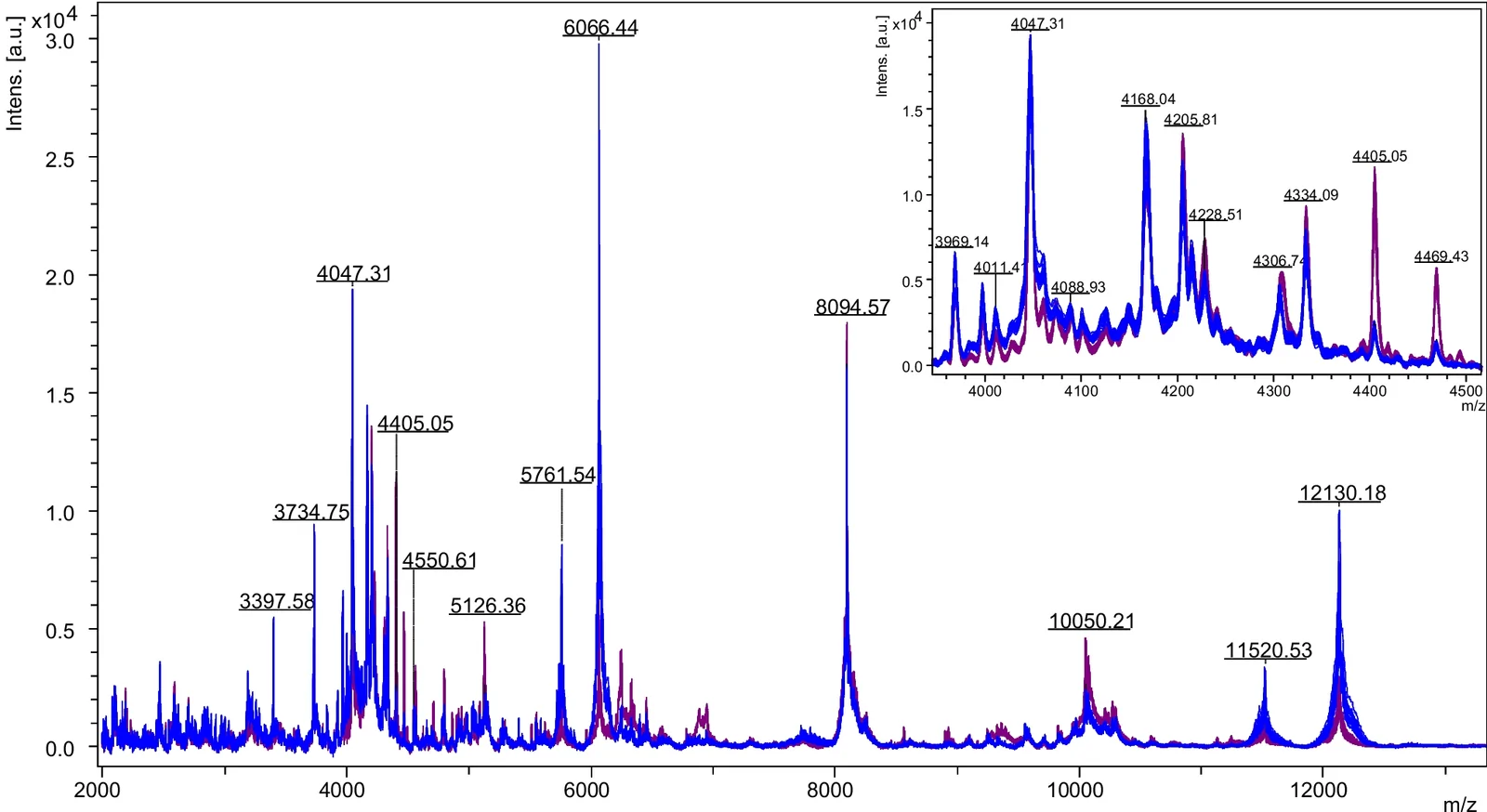
Comparison of the influence of homogenization on the MALDI-TOF MS spectra of a *D. reticulatus* tick (third replicate). Left four legs (blue) were handled with the new workflow as stated in the methods section. Right four legs (purple) were additionally ground with a plastic micropistill in the reaction tube

**Fig. 13:**
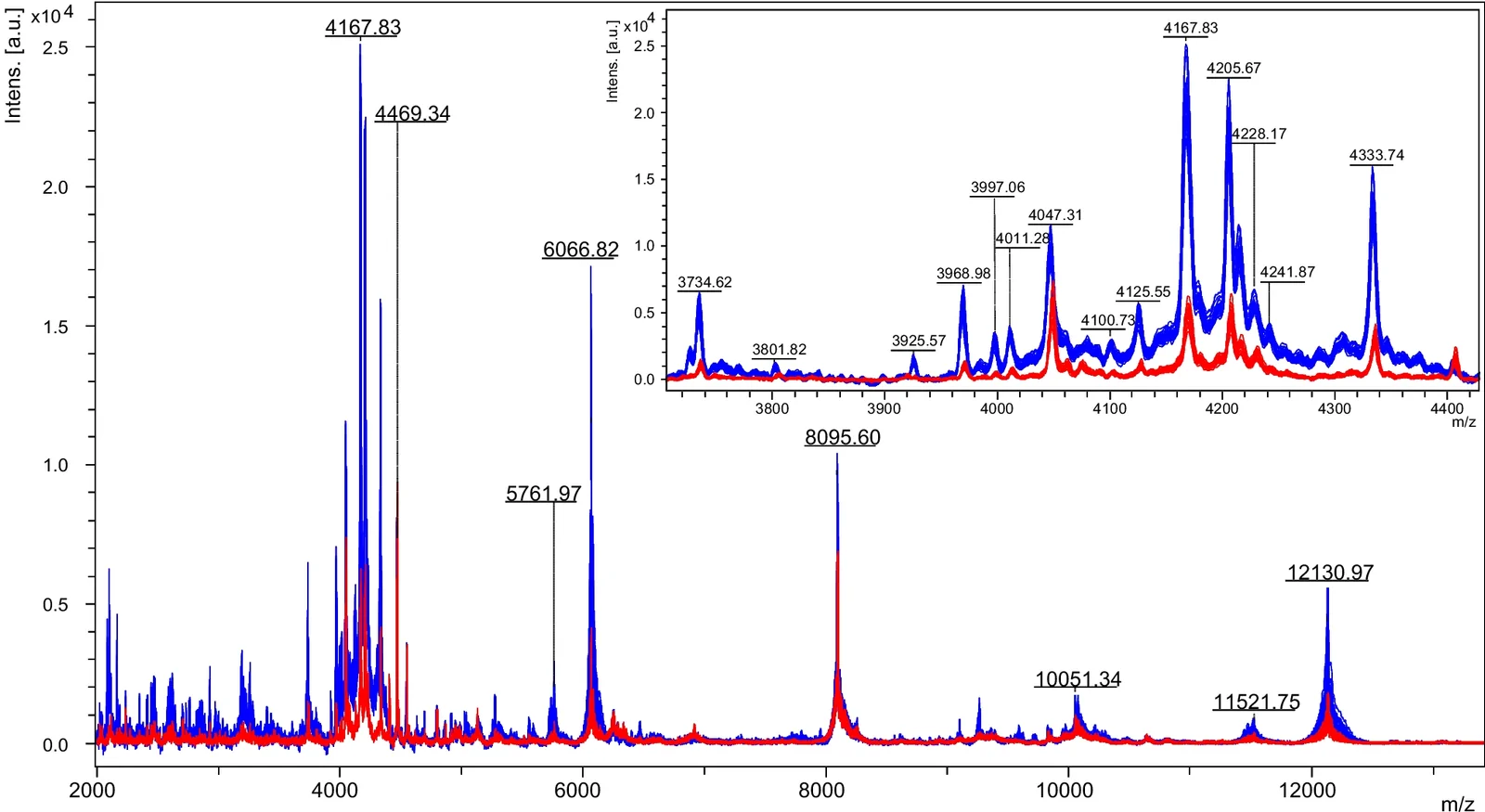
Comparison of the MALDI-TOF MS spectra of a *D. reticulatus* tick (second replicate). Left legs were processed with the new workflow (blue) and right legs with the recently published procedure by Beltran et al. (2023) (red)

**Fig. 14:**
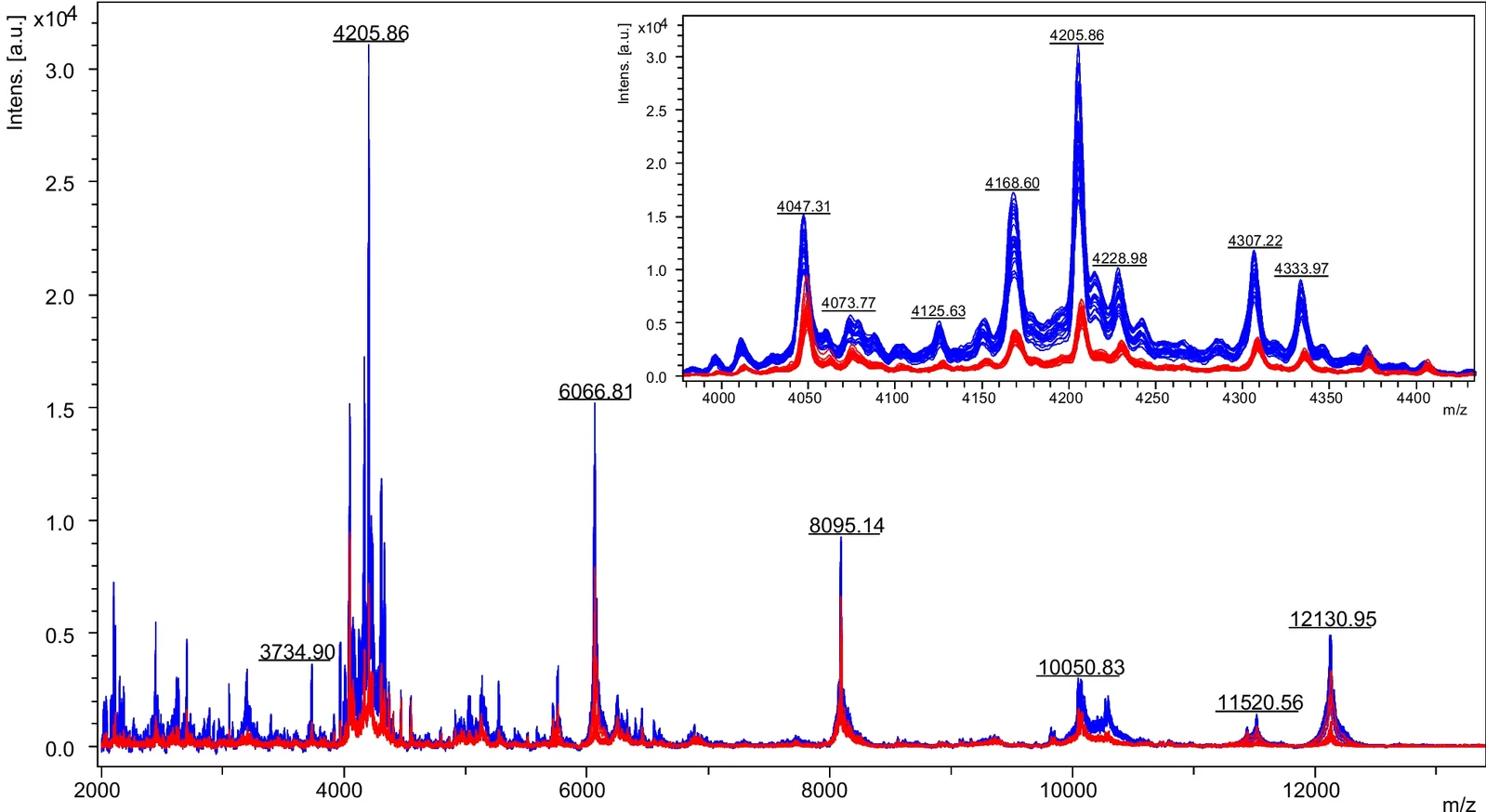
Comparison of the MALDI-TOF MS spectra of a *D. reticulatus* tick (third replicate). Left legs were processed with the new workflow (blue) and right legs with the recently published procedure by Beltran et al. (2023) (red)

**Fig. 15:**
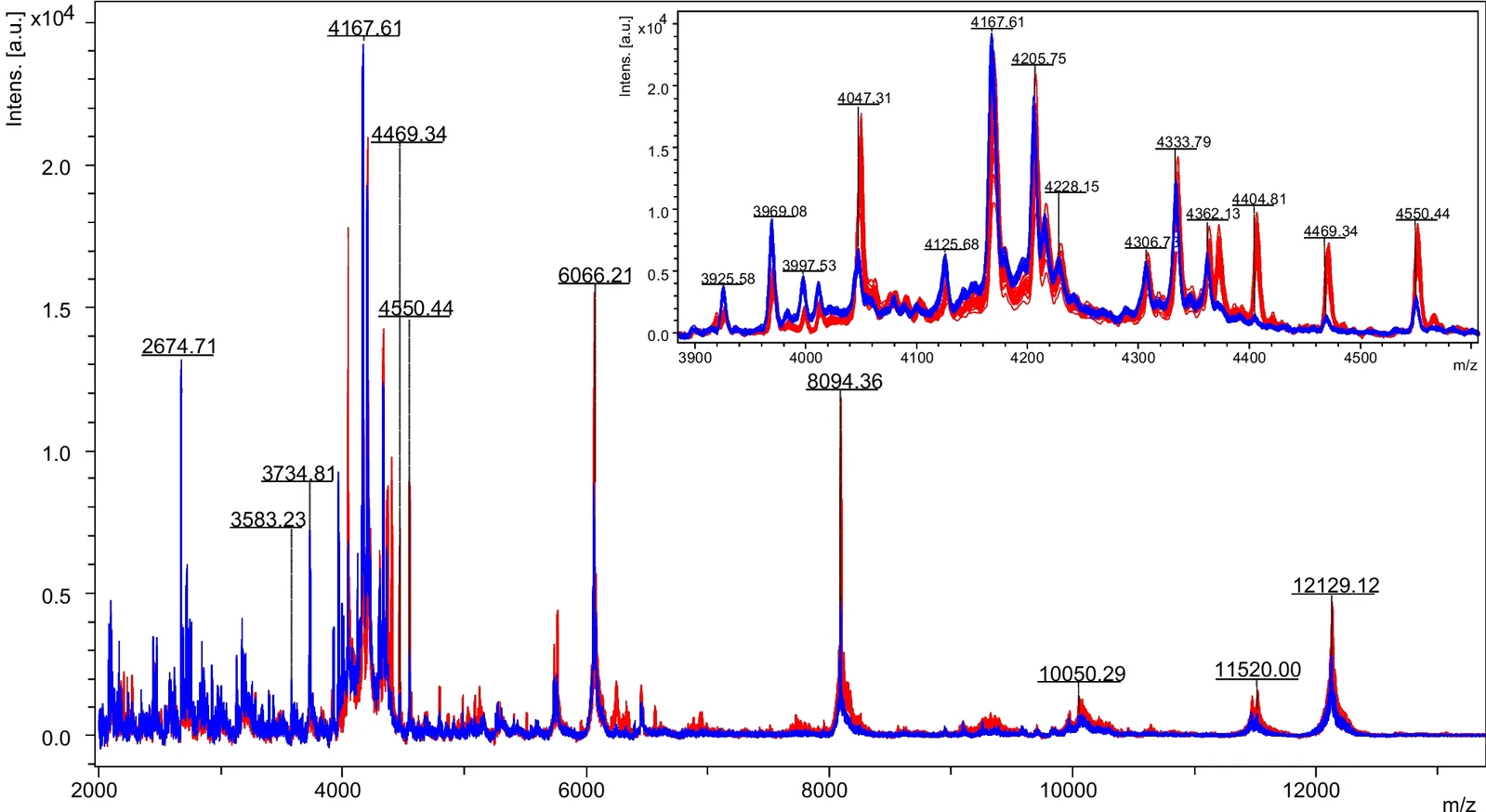
Comparison of the MALDI-TOF MS spectra of a *D. reticulatus* tick (second replicate). Left legs were processed with the new workflow (blue) and right legs with the recently published procedure by Galletti et al. (2024) (red)

**Fig. 16:**
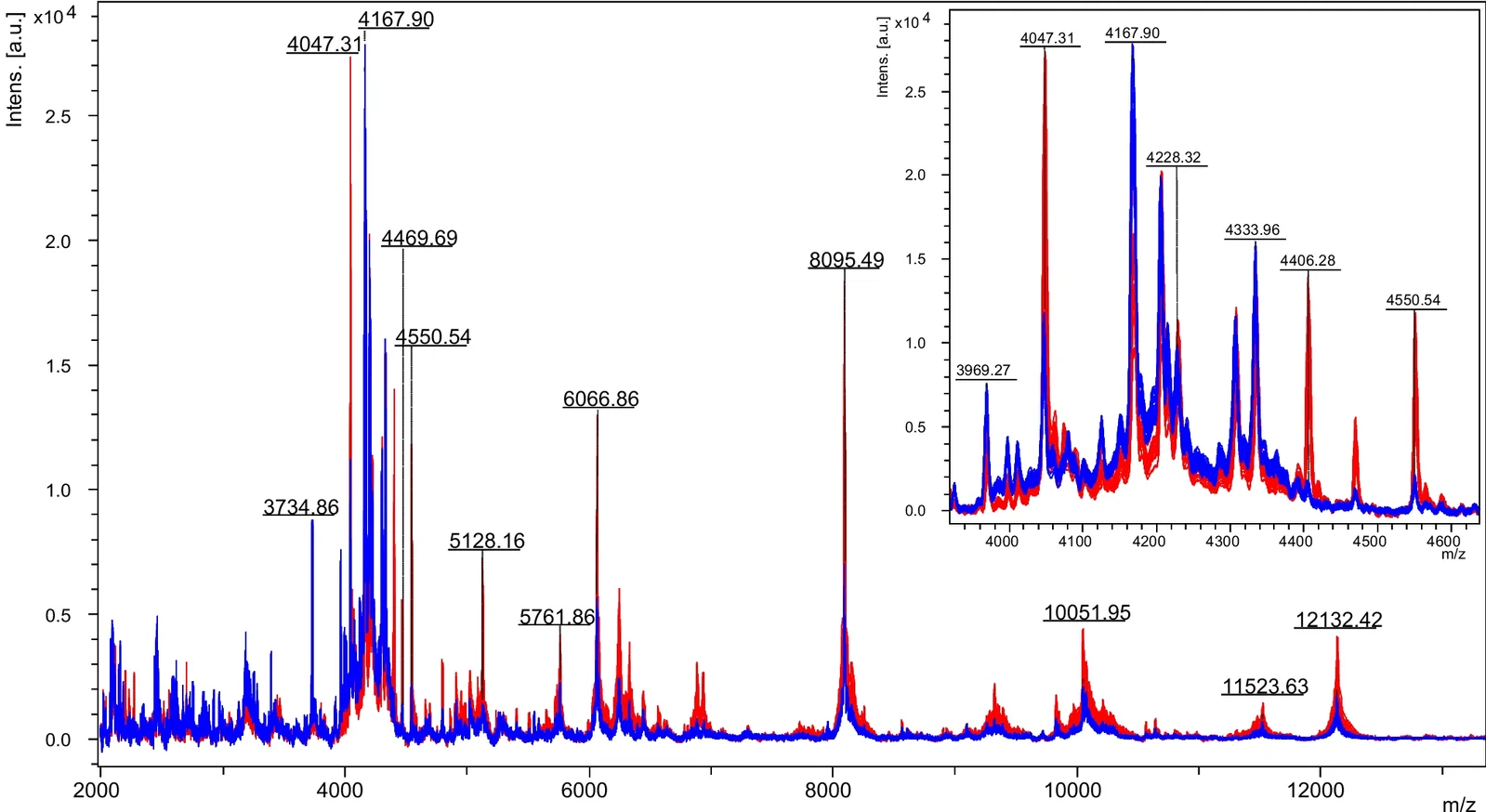
Comparison of the MALDI-TOF MS spectra of a *D. reticulatus* tick (third replicate). Left legs were processed with the new workflow (blue) and right legs with the recently published procedure by Galletti et al. (2024) (red)

**Fig. 17:**
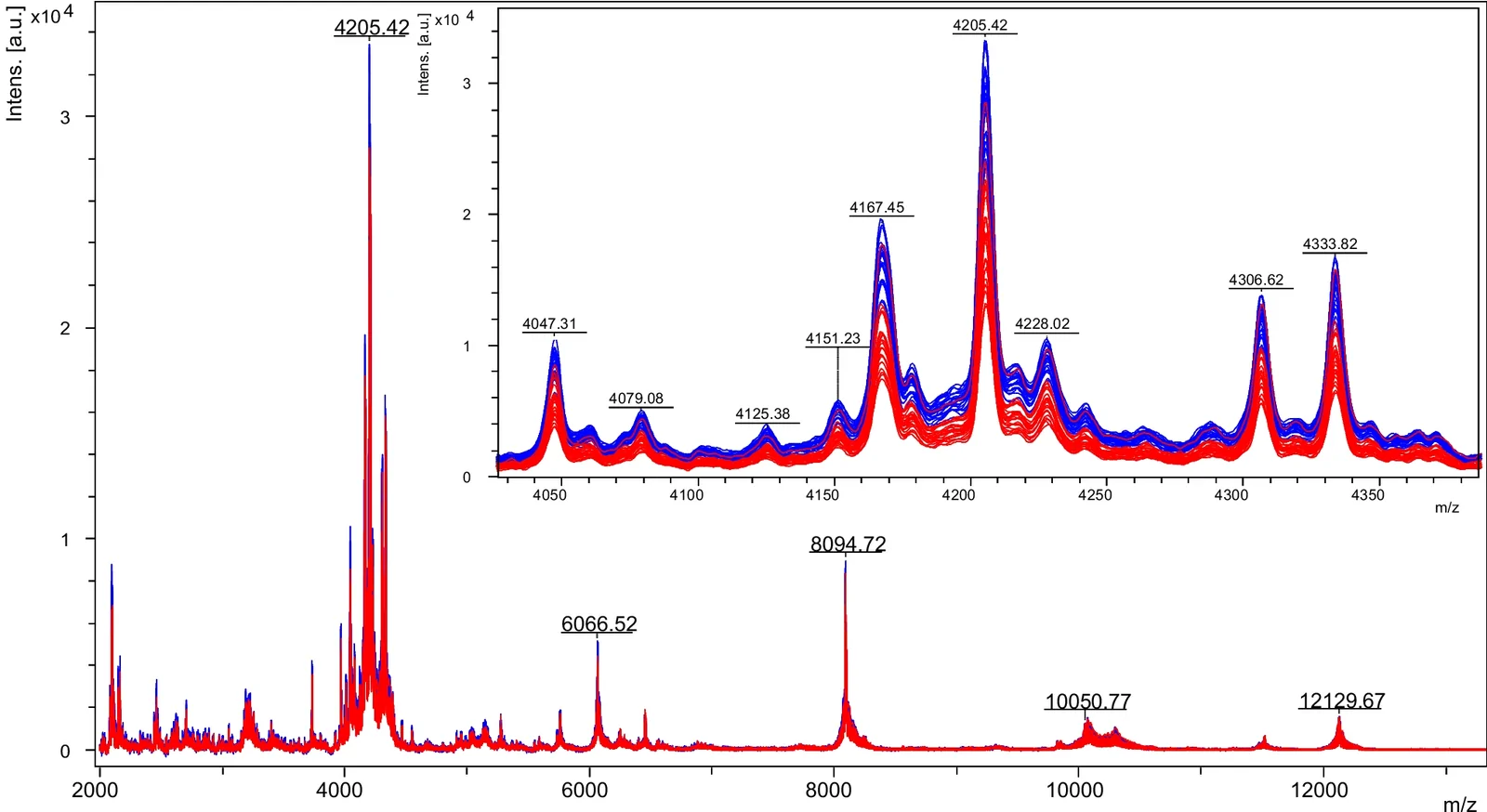
Comparison of the Bruker acquisition methods MBT_AutoX and MBT_AutoX_FilFungi_2 with tick samples. The exact same eight spots of a *D. reticulatus* tick sample (second replicate) was measured with the MBT_AutoX (red) and MBT_AutoX_FilFungi_2 method (blue)

**Fig. 18:**
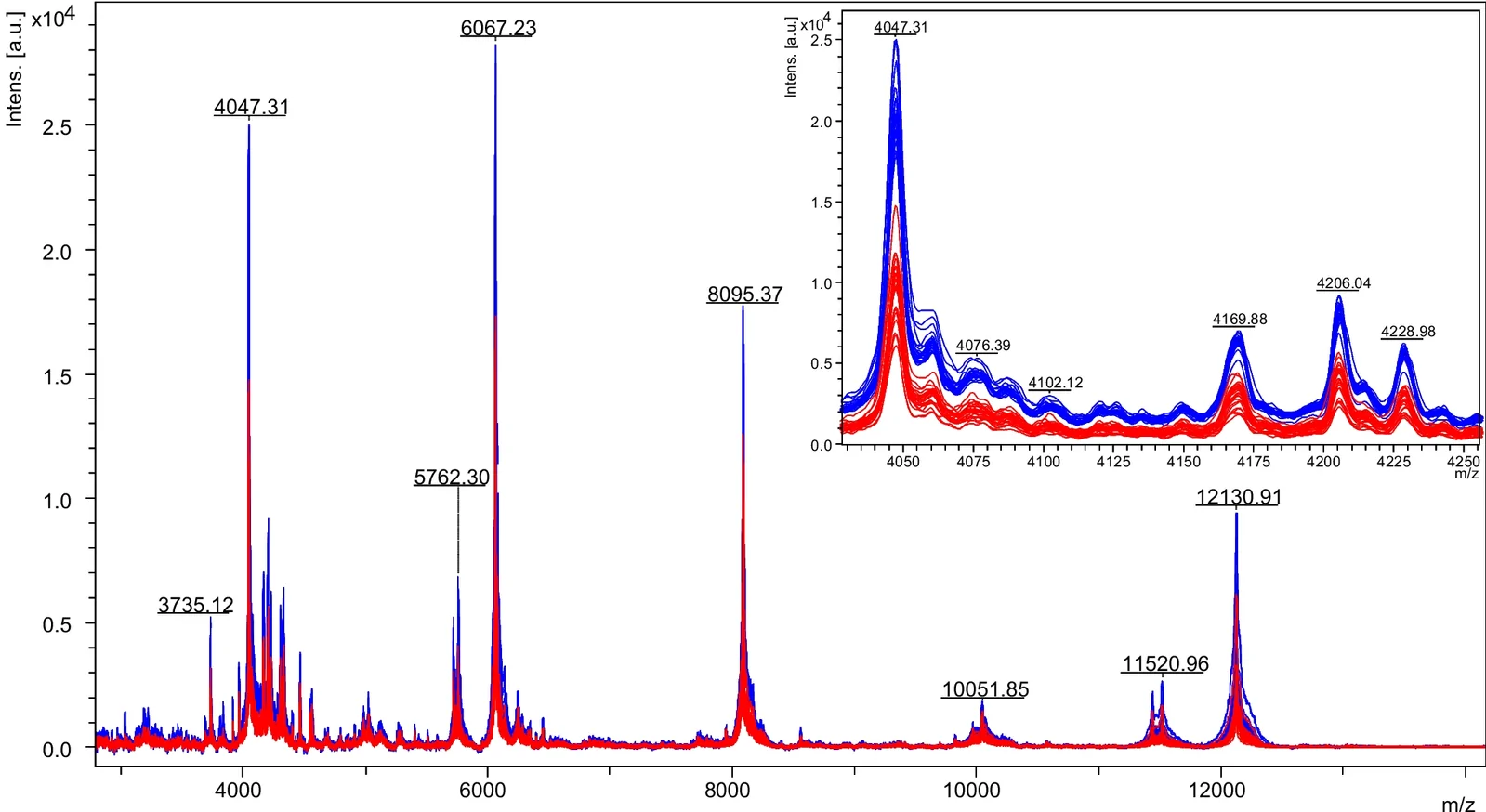
Comparison of the Bruker acquisition methods MBT_AutoX and MBT_AutoX_FilFungi_2 with tick samples. The exact same eight spots of a *D. reticulatus* tick sample (third replicate) was measured with the MBT_AutoX (red) and MBT_AutoX_FilFungi_2 method (blue)

**Fig. 19:**
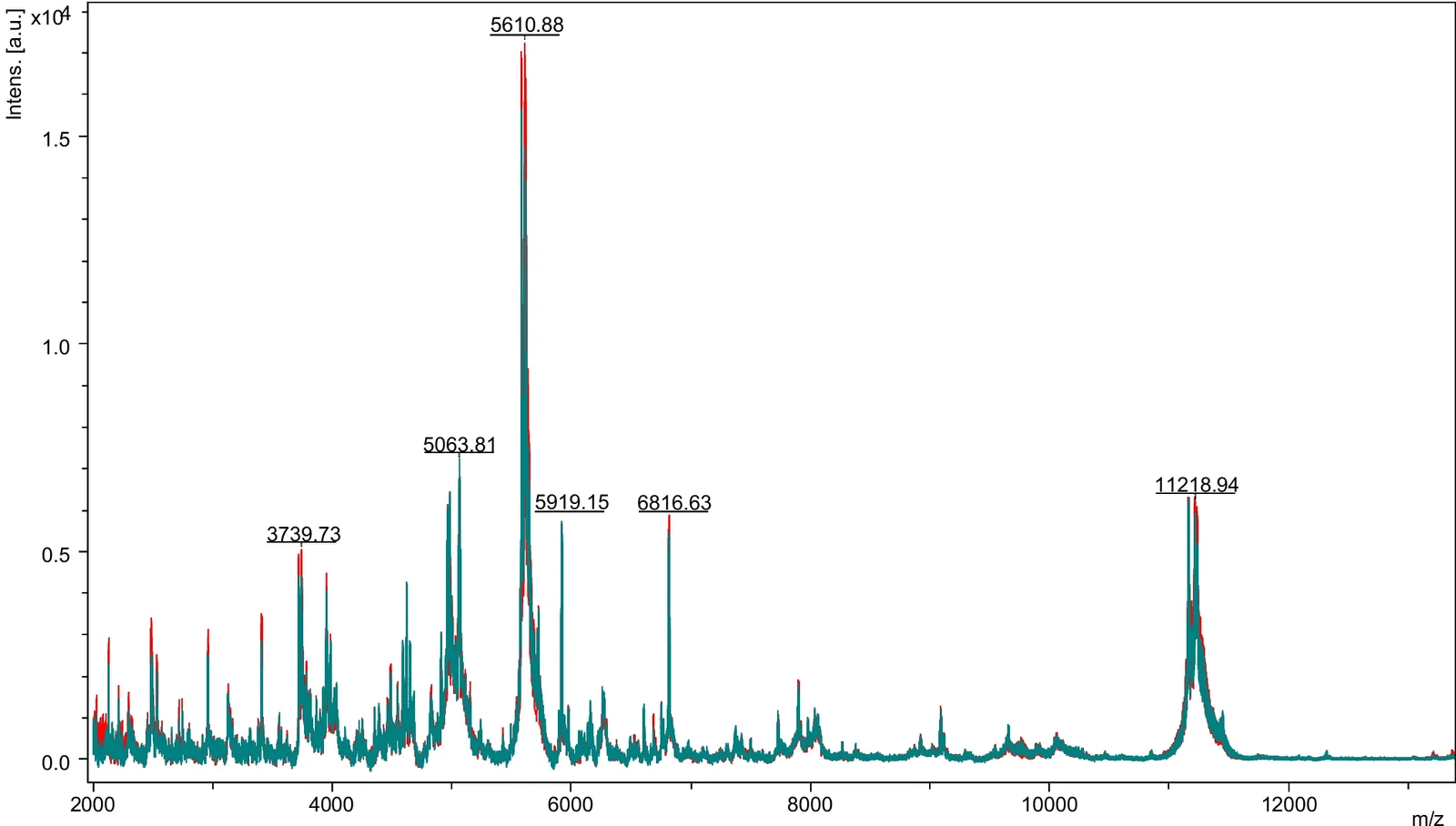
Comparison of different target types with the spectra derived of a male, adult *I. persulcatus* tick. The same extract was spotted and measured on a single-use Bruker MBT Biotarget 96 plate (red) and on Bruker MSP 96 polished steel BC targets (green)

**Fig. 20:**
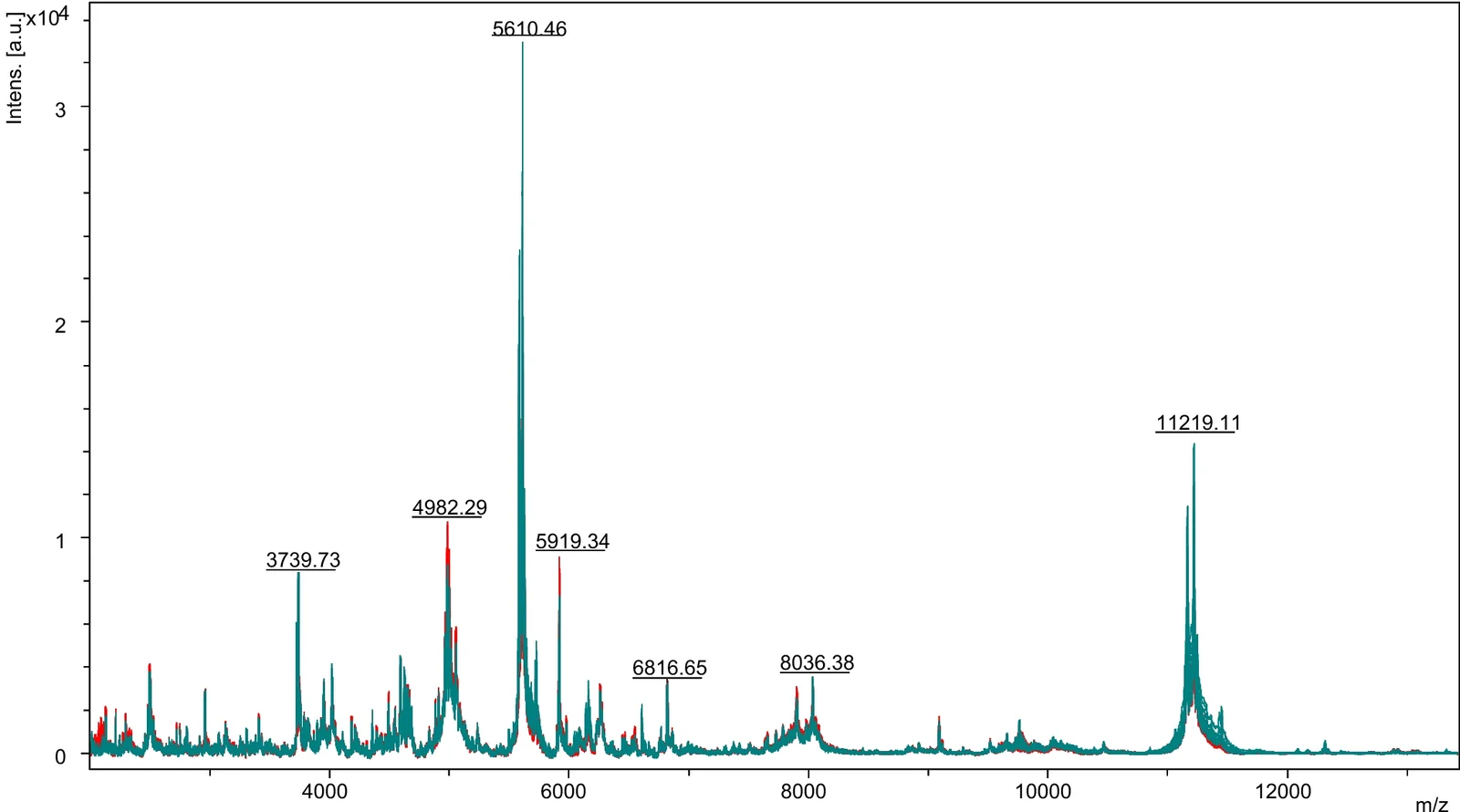
Comparison of different target types with the spectra derived of a male, adult *I. persulcatus* tick. The same extract was spotted and measured on a single-use Bruker MBT Biotarget 96 plate (red) and on Bruker MSP 96 polished steel BC targets (green)

**Fig. 21:**
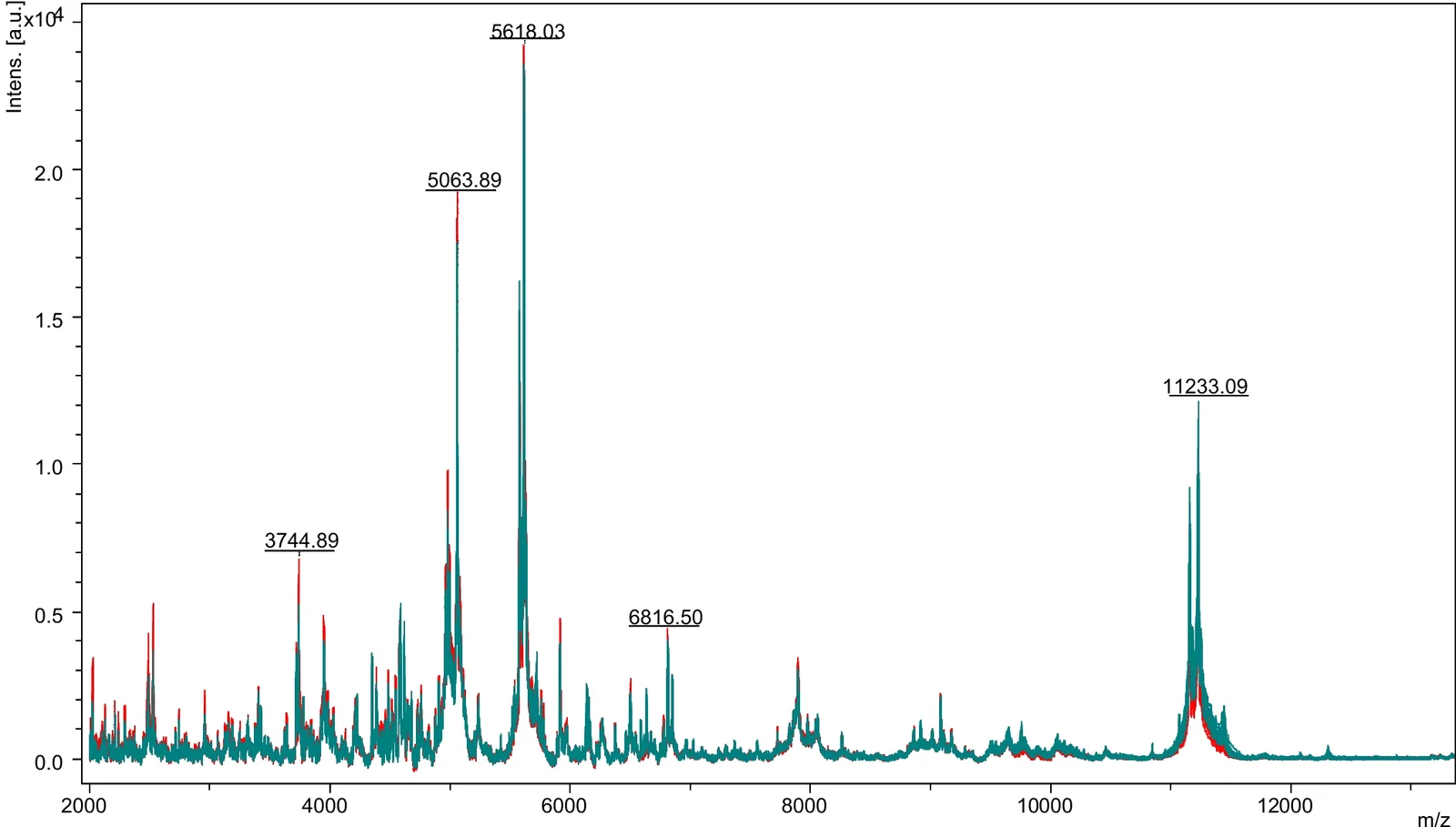
Comparison of different target types with the spectra derived of a male, adult *I. persulcatus* tick. The same extract was spotted and measured on a single-use Bruker MBT Biotarget 96 plate (red) and on Bruker MSP 96 polished steel BC targets (green)

Figures 2, 3, 4, 5, 9, 10, 11, 12, 13, 14, 15 and 16 (partly in appendix) clearly show that not only do the left and right four tick legs produce identical reproducible spectra (Figs. 2, 9 and 10), but also that the newly established, economically and qualitatively optimized method definitely does not exhibit any loss of needed information compared to additional homogenization (Figs. 3, 11 and 12) and further not to previously published methods from Beltran et al. (2023) (Figs. 4, 13 and 14) and Galletti et al. (2024) (Figs. 5, 15 and 16). The new improved sample extraction and processing workflow may replace protocols using homogenization and none of the mentioned optimizations for the identification of ticks due to its result quality and parallel time saving around 90% per tick compared to Galletti et al. (2024) and around 30% for two ticks compared to Beltran et al. (2023). Further optimized and reproducible MALDI-TOF MS spectra derived from the new workflow will build the fundament for the creation of a new commercially available MALDI-TOF MS reference library which might push tick identification to a new level. Different approaches for optimizations of the actual identification process of unknown tick samples against the new library will be further tested.

## Data Availability

The raw data supporting the conclusions of this article, will be made available to qualified researchers by the authors upon reasonable request.
